# Transplantation of endothelial progenitor cells attenuates pulmonary fibrosis via IFITM3-containing exosomes

**DOI:** 10.1172/jci.insight.199743

**Published:** 2026-10-08

**Authors:** Ying-Wei Lan, Jonathan Do, Wen Gao, Zicheng Deng, Xiaomei Xia, Enhong Li, Jillian Kash, Gautam Verma, Angara Sureshbabu, Kenneth S. Knox, Ross M. Bremner, Nicholas E. Banovich, Vladimir V. Kalinichenko, Tanya V. Kalin

**Affiliations:** 1 Phoenix Children’s Research Institute, Department of Child Health, University of Arizona College of Medicine–Phoenix, Phoenix, Arizona, USA.; 2 Norton Thoracic Institute, St. Joseph’s Hospital and Medical Center, Dignity Health, Phoenix, Arizona, USA.; 3 Department of Internal Medicine, Division of Pulmonary Medicine, University of Arizona College of Medicine–Phoenix, Phoenix, Arizona, USA.; 4 Division of Bioinnovation and Genome Sciences, The Translational Genomics Research Institute (TGen), Phoenix, Arizona, USA.; 5 Division of Neonatology and; 6 Center for Cancer and Blood Disorders, Phoenix Children’s Hospital, Phoenix, Arizona, USA.

**Keywords:** Pulmonology, Vascular biology, Endothelial cells, Fibrosis, Therapeutics

## Abstract

Endothelial progenitor cells (EPCs) are critical for vascular regeneration after injury. However, their role in pulmonary fibrosis (PF) remains unclear. Here, we identified a previously unrecognized population of lung capillary EPCs coexpressing c-KIT and FOXF1 (capEPCs) in adult human and mouse lungs. capEPCs were significantly reduced in lungs from patients with PF and in bleomycin-injured mice, implicating loss of this regenerative endothelial population in disease pathogenesis. Transplantation of donor capEPCs attenuated experimental PF, improved survival, reduced collagen deposition, and restored lung function. Donor capEPCs engrafted into the lung microcirculation of bleomycin-injured mice. Single-cell RNA sequencing identified interferon-induced transmembrane protein 3 as a highly enriched transcript in capEPCs. Conditioned media from IFITM3-overexpressing EPCs or recombinant IFITM3 enhanced endothelial proliferation and angiogenesis while suppressing TGF-β1–induced fibroblast activation in vitro. IFITM3 was found in capEPC-derived exosomes. Treatment with IFITM3-containing exosomes recapitulated the therapeutic effects of capEPC transplantation by improving endothelial function, inhibiting fibroblast activation, increasing animal survival, reducing lung fibrosis, and restoring lung function. Together these findings identified capEPC deficiency as a feature of PF and demonstrated that IFITM3-containing exosomes promoted vascular repair. Restoration of capEPC regenerative function or delivery of IFITM3-enriched exosomes may represent a promising therapeutic strategy for human PF.

## Abstract 2

We have identified new lung endothelial progenitors (EPC) that decline during fibrotic lung injury. Transplantation of EPC or delivering their exosomes prevent lung fibrosis.

## Graphical abstract

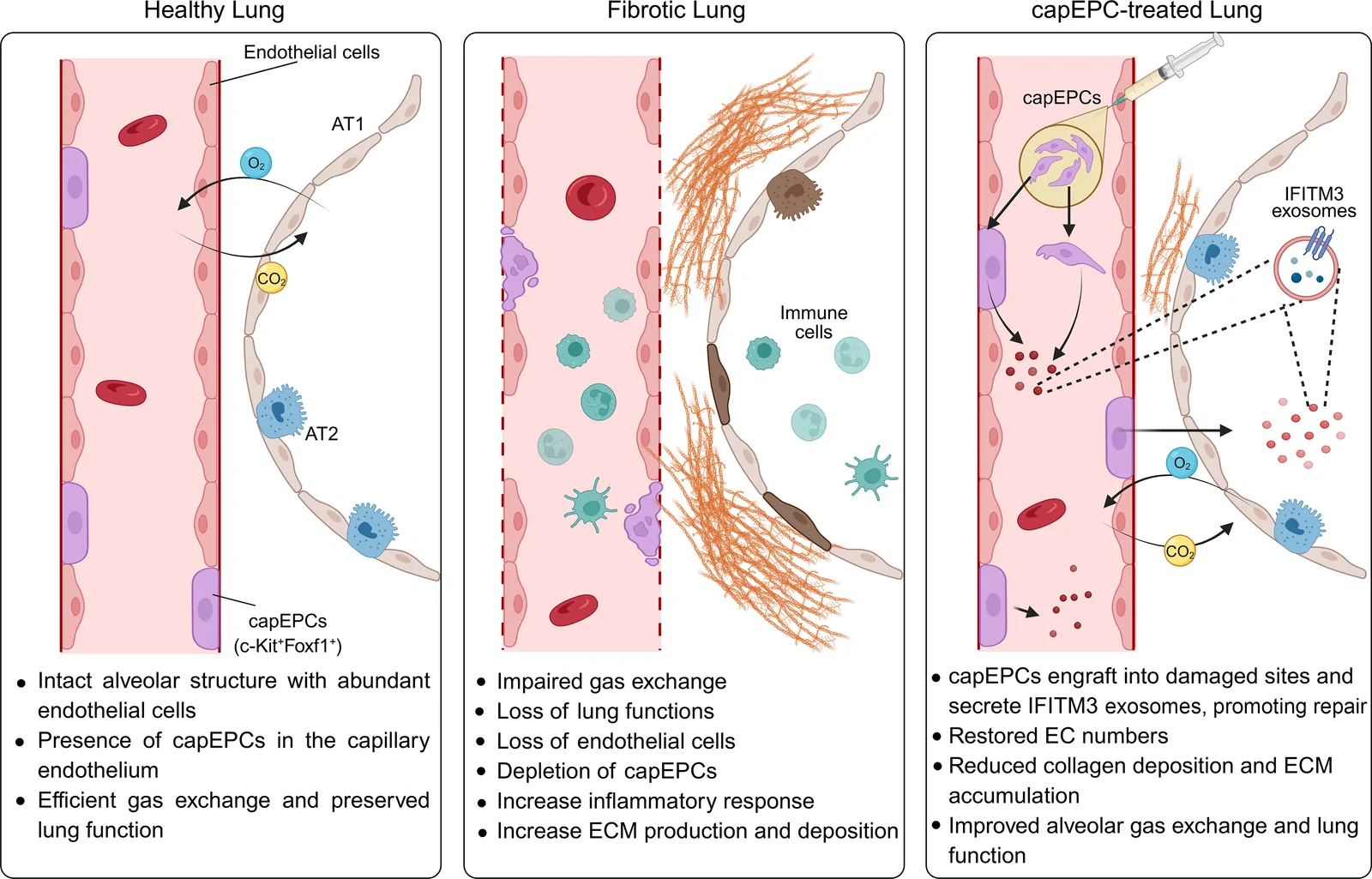

## Introduction

Aberrant repair following lung injury is a central driver of pulmonary fibrosis (PF) (1). Idiopathic pulmonary fibrosis (IPF), the most severe form of PF, is a progressive interstitial lung disease characterized by irreversible fibrotic remodeling, declining lung function, and limited therapeutic options (2, 3). The pathophysiology of IPF includes repetitive alveolar epithelial injury, which, in the presence of various risk factors, results in the loss of type I alveolar epithelial cells (AT1 cells), accumulation of transitional AT1/AT2 cells, abnormal activation of fibroblasts and immune cells, and the loss of alveolar microvasculature (4, 5).

Multiple cell types are involved in the development of PF, including pulmonary endothelial cells (ECs) that are critical for gas exchange function, maintenance of vascular homeostasis, and integrity of the alveolar-capillary barrier (6). The dysfunctions of lung endothelium can directly and indirectly modulate the behavior of surrounding cells through the secretion of various mediators. The reduced secretion of sphingosine-1-phosphate (S1P) from ECs inactivates the YAP-induced differentiation of AT2 cells into AT1 cells in response to lung injury (7). The elevated levels of adhesion molecules, such as ICAM-1, VCAM-1, and selectin that are secreted by ECs, correlated with enhanced immune cell recruitment and extravasation in the fibrotic lung (8). The presence of profibrotic mediators, such as TGF-β and ET-1, led to downregulation of BMPR2 in ECs, fibroblasts, and smooth muscle cells to facilitate vascular remodeling and fibrosis progression (9). Because of the close anatomic proximity between alveolar epithelium and capillary ECs, the initial epithelial injury also affects pulmonary endothelium, leading to increased vascular permeability, aberrant angiogenesis, partial loss of microvasculature, and impaired EC repair (6, 10, 11). The specific mechanisms driving the repair of damaged ECs in PF are not well characterized.

Repair of pulmonary endothelium has been recently shown to be dependent on endothelial progenitor cells (EPCs) (12). The number of EPCs is decreased in fibrotic lungs and is associated with reduced total lung capacity, impaired alveolo-arterial diffusion, and worse prognosis (13, 14). Restoring the number of EPCs and their functions may provide a promising therapeutic approach to promote normal lung repair without inducing PF. EPCs are classified based on their developmental origin and anatomical localization, including tissue-resident EPCs, distal and proximal EPCs, circulating EPCs, and clonal repopulating endothelial cells (15–17). Different EPCs have distinct markers and functions (12, 18).

In the distal lung, general capillary ECs (CAP1) were shown to act as progenitor cells giving rise to aerocytes (CAP2) that perform the critical gas exchange function (19, 20). Within microvasculature of neonatal lung, a unique population of KIT^+^FOXF1^+^ EPCs has been recently identified (21, 22). Using neonatal mouse models of alveolar capillary dysplasia with misalignment of pulmonary veins (ACDMPV) and bronchopulmonary dysplasia (BPD), it has been shown that the decreased number of KIT^+^FOXF1^+^ EPCs was associated with underdeveloped lung microvasculature and impaired alveologenesis (23). Adoptive transfer of neonatal KIT^+^FOXF1^+^ EPCs stimulated lung angiogenesis and prevented alveolar simplification in mouse ACDMPV and BPD models (21–24). While the importance of neonatal KIT^+^FOXF1^+^ EPCs has been established for neonatal lung diseases, it is unknown whether these cells exist in the adult human and mouse lungs and whether these cells have the potential to improve lung tissue repair and to prevent fibrotic remodeling after lung injury.

In this study, we identify a previously unrecognized population of adult KIT^+^FOXF1^+^ capillary EPCs (capEPCs) in human and mouse lungs. We demonstrate that capEPCs are depleted during PF and that transplantation of donor capEPCs into bleomycin-injured mice restores lung function, improves survival, and attenuates fibrosis through IFITM3-containing exosomes. These findings identify capEPC-mediated vascular repair as a previously unrecognized feature of PF and identify capEPC-derived exosomes as a promising therapeutic strategy for pulmonary fibrosis.

## Results

### Adult lung capEPCs localize within CAP1 and are decreased in fibrotic lungs of PF patients.

Since KIT^+^FOXF1^+^ EPCs were originally identified in embryonic and neonatal lungs (22–24), we next determined whether these EPCs exist in adult lungs. We analyzed publicly available single-cell RNA sequencing (scRNA-seq) datasets of human lung tissue from 66 patients with PF and 48 unaffected donors (25). The samples from PF patients were stratified into 2 groups, “less fibrotic” and “more fibrotic,” based on the differentially affected areas of one lung used to prepare sequencing libraries (25). Endothelial cells were reclustered using EC-specific marker genes (26), identifying 5 distinct endothelial subclusters (Figure 1A and Supplemental Figure 1, A and B; supplemental material available online with this article; https://doi.org/10.1172/jci.insight.199743DS1). Consistent with previously published data (27), the percentage of CAP1, CAP2, arterial ECs, and venous ECs was decreased, while that of systemic venous ECs was increased, in PF lungs compared with donor lungs (Supplemental Figure 1C). Double-positive KIT^+^FOXF1^+^ EPCs (referred to as capEPCs hereafter) were mostly present within the CAP1 subcluster (Figure 1A) and had upregulated signaling pathways important for progenitor cells, including pluripotent stem cell differentiation, KIT, IGF-1, and FGF-1 signaling (Supplemental Figure 1D). The percentage of capEPCs was reduced in the lungs of PF patients, including both “less fibrotic” and “more fibrotic” PF, compared with donor lungs, suggesting that the loss of capEPCs is an early event in PF progression (Figure 1, B and C, and Supplemental Figure 1F). Next, we used immunostaining with CD31, FOXF1, and KIT antibodies and demonstrated that the abundance of capEPCs was decreased in the lungs of PF patients (Figure 1D). Compared with donor lungs, transcriptional signatures of capEPCs from PF lungs showed decreased gene signatures important for cell survival, proliferation, and progenitor functions, including NRF2, JAK-STAT, PDGFB, KIT, MAPK, and IGF-1 signaling pathways (Figure 1E and Supplemental Figure 1E). In contrast, profibrotic and proinflammatory pathways, such as TGF-β, IL-2, p53, and IL-6 pathways, were upregulated in fibrotic capEPCs (Figure 1E). Altogether, capEPCs are present in the human adult lung and belong to the CAP1 ECs, the number of capEPCs is decreased in lungs of PF patients, and their transcriptional signature is changed in PF.

### Mouse lung capEPCs localize within CAP1 and are decreased in lungs of bleomycin-treated mice.

We next determined whether capEPCs existed in the adult mouse lung. Using publicly available scRNA-seq (28), we reclustered ECs to identify 4 distinct subclusters (Figure 2A and Supplemental Figure 2, A and B). Consistent with human data, the mouse capEPCs were identified within the CAP1 subcluster of ECs (Figure 2, A–C) and had upregulated signaling pathways important for progenitor cells (Supplemental Figure 2C). The abundance of capEPCs was decreased in the lungs of bleomycin-treated mice compared with control mouse lungs (Figure 2, B and C, and Supplemental Figure 2E). The numbers of capEPCs were decreased as early as day 7 after bleomycin injury and remained reduced during fibrotic stages (days 10–21) based on the analysis of the publicly available scRNA-seq dataset (GEO GSE141259) (29) (Supplemental Figure 2F). The decrease in the capEPC abundance correlated with decreased vascular gene module score and increased fibrosis gene module score (Supplemental Figure 2G). No changes in the numbers of KIT^+^CD45^+^ cells were shown between control and bleomycin-treated lungs (Supplemental Figure 2H). Consistent with human data, the transcriptional signature of capEPCs was changed during fibrotic remodeling in bleomycin-treated mice (Figure 2D). Specifically, capEPCs within fibrotic mouse lungs exhibited decreased signaling pathways important for cell survival, proliferation, and progenitor functions, including VEGF signaling, IGF signaling, PI3K/Akt signaling, and Ras signaling (Figure 2D). As in human capEPCs, profibrotic and proinflammatory signatures were increased in murine capEPCs from bleomycin-injured lungs (Figure 2D and Supplemental Figure 2D). Immunostaining of lung tissue sections with CD31, FOXF1, and KIT antibodies demonstrated that capEPCs were abundantly present in the normal mouse lung but decreased in the bleomycin-treated lungs (Figure 2E and Supplemental Figure 3). To quantify the percentage of capEPCs within lung ECs, we next used Foxf1-GFP reporter mice, in which GFP was knocked into the endogenous Foxf1 gene locus (24) (Figure 2F). Flow cytometry analysis demonstrated that the total number of lung ECs and the percentage of capEPCs within ECs were decreased in the bleomycin-treated lungs of Foxf1-GFP reporter mice compared with untreated control lungs at day 21 after 2 bleomycin administrations (Figure 2F). Altogether, capEPCs are present in the adult mouse lung and decreased after bleomycin injury. Strong similarities in capEPC signatures indicate common signaling mechanisms in human and mouse capEPCs during fibrotic lung remodeling.

### Transplantation of capEPCs improves mouse survival and lung functions in a preclinical model of PF.

Since the number of capEPCs is decreased during PF and their transcriptomic signatures are conserved in humans and mice, we next tested whether the transplantation of donor capEPCs to recipient fibrotic lungs would have the therapeutic benefit. To lineage-label donor cells, we crossed Foxf1-GFP reporter mice with transgenic mice expressing ubiquitous tdTomato (Figure 3A). The double-transgenic donor mice were used for FACS sorting of FoxF1^+^KIT^+^ capEPCs from adult lungs. As a control for cell transplantation, we used adult FoxF1^–^KIT^+^ ECs that show no progenitor properties in neonatal lungs (24). The donor cells were transplanted intravenously to bleomycin-treated recipient mice that had already developed fibrosis and had shown measurable collagen deposition at day 8 after 2 intratracheal administrations of bleomycin (30) (Figure 3A). The other control included bleomycin-treated recipient mice that were mock-transplanted (treated with saline instead of cells).

Mice transplanted with capEPCs exhibited markedly improved survival, with all mice surviving in this group, which was a better survival outcome than that of mock-transplanted mice or mice transplanted with the control FoxF1^–^KIT^+^ ECs (Figure 3B). We next assessed body weight changes in response to bleomycin treatment. Transplantation of capEPCs improved recovery of body weight loss in fibrotic mice by day 21 compared with both controls (Figure 3C). Furthermore, transplantation of capEPCs improved the peripheral arterial blood oxygenation measured by oximeter (Figure 3D), as well as lung mechanics measured by flexiVent (Figure 3, E and F). Respiratory system resistance (R_rs_) was decreased, whereas lung inspiratory capacity and lung compliance were increased, in the capEPC-transplanted group compared with other bleomycin-injured groups (Figure 3, E and F). Thus, transplantation of capEPCs increases mouse survival, promotes body weight recovery, and improves respiratory functions after fibrotic bleomycin injury.

### Transplantation of capEPCs attenuates fibrotic remodeling in bleomycin-treated mice.

To further assess the therapeutic benefits of capEPC transplantation in a mouse model of PF, we compared fibrotic remodeling and collagen deposition in the lungs of bleomycin-injured mice transplanted with either capEPCs or control FoxF1^–^KIT^+^ ECs. Transplantation of capEPCs reduced Ashcroft score (Figure 4A) and decreased collagen deposition assessed by Masson’s trichrome staining (Figure 4B), Sirius red/fast green staining (Figure 4C), and immunostaining for αSMA (Figure 4D). Quantitative hydroxyproline assay demonstrated the decreased collagen levels in the lungs of capEPC-transplanted mice compared with controls (Figure 4E). Consistent with decreased fibrotic remodeling after capEPC transplantation, *Col1a1*, *Col3a1*, *Fn1*, and *Acta2* mRNAs were reduced in total lung RNA (Figure 4, F–I). Moreover, capEPC transplantation successfully restored the *Epcam* and *Cdh1* mRNAs compared with control treatments, suggesting improvement in the lung epithelium (Supplemental Figure 4A). The CTHRC1^+^ fibroblasts were decreased in the capEPC-treated group (Supplemental Figure 4B). No changes in the numbers of inflammatory cells were found in capEPC-transplanted and control lungs using flow cytometry analysis (Supplemental Figure 4C). Thus, transplantation of donor capEPCs effectively reduced fibrotic lung remodeling and collagen deposition in the bleomycin-injured recipient mice.

### Transplantation of capEPCs increases the number of lung ECs in bleomycin-injured mice.

Since the number of capillary ECs is decreased in mouse and human fibrotic lungs (30), we examined EC numbers after capEPC transplantation. Immunofluorescent imaging and FACS analysis of lung tissue demonstrated an increase in the number of ECs in the lungs of bleomycin-injured mice transplanted with capEPCs (Figure 5, A and B). The mean fluorescence intensity of CD31 staining in ECs was increased in the capEPC-transplanted group, indicating that transplantation of capEPCs not only increased the number of CD31^+^ ECs but also increased CD31 cell surface expression in these cells (Figure 5B). Furthermore, more proliferating ECs and fewer apoptotic ECs were found in the lungs of bleomycin-injured mice transplanted with capEPCs (Supplemental Figure 5, A and B). Next, quantitative reverse transcription PCR (qRT-PCR) analysis of total lung tissue demonstrated that transplantation of capEPCs restored the expression of endothelial cell–specific *Cdh5*, *Pecam1*, and *Kdr* mRNAs as well as expression of *Foxf1* (Figure 5C), which is consistent with the increased numbers of lung ECs (Figure 5, A and B).

Finally, since the donor capEPCs were labeled with tdTomato, we assessed whether capEPCs engrafted into the lung endothelium of the bleomycin-injured mice. Immunostaining for CD31 and tdTomato showed a higher percentage of tdTomato^+^ capEPCs present within CD31^+^ ECs in comparison with the mice transplanted with control Foxf1^–^Kit^+^ ECs (Figure 5D). Flow cytometry analysis further supported these findings, detecting approximately 16% tdTomato^+^ ECs in the lungs of capEPC-transplanted mice 2 weeks after transplantation, compared with only 3% tdTomato^+^ ECs in the lungs of control mice (Figure 5E). These data indicate better engraftment efficiency of donor capEPCs to the recipient lung, compared with control Foxf1^–^Kit^+^ ECs.

Since the increased engraftment efficiency of donor capEPCs may still be insufficient to explain the robust therapeutic effect of capEPC transplantation, we next determined whether capEPCs mediate antifibrotic effects via paracrine signals to recipient cells.

### IFITM3 is a direct transcriptional target of FOXF1.

To identify molecular mechanisms underlying antifibrotic effects of capEPCs, we compared the transcriptome of capEPCs and control Foxf1^–^Kit^+^ ECs using the scRNA-seq dataset of adult mouse lungs. High *Foxf1* mRNA levels in capEPCs and the absence of *Foxf1* in control Foxf1^–^Kit^+^ ECs were confirmed using violin plot (Figure 6A). We next identified differentially expressed genes between capEPCs and control Foxf1^–^Kit^+^ ECs. One of the highly differentially expressed genes in capEPCs was interferon-induced transmembrane protein 3 (*Ifitm3*) (Figure 6B). The high level of *Ifitm3* mRNA in capEPCs was confirmed by qRT-PCR using FACS-sorted capEPCs and Foxf1^–^Kit^+^ ECs from the lungs of Foxf1-GFP reporter mice (Figure 6C). Next, we used the human donor lung scRNA-seq dataset to compare *IFITM3* expression in human capEPCs and FOXF1^–^KIT^+^ ECs. *IFITM3* mRNA was increased in human capEPCs, correlating with *FOXF1* expression (Figure 6D). In bronchoalveolar lavage fluid (BALF) from patients with idiopathic pulmonary fibrosis (IPF), IFITM3 protein levels were significantly reduced in comparison with healthy controls, as reported in a quantitative BALF proteomics study (31) (Figure 6E). Knockdown of *Foxf1* in Kit^+^ ECs using siRNA decreased *Ifitm3* mRNA and reduced angiogenesis in vitro (Figure 6, F and G). Finally, using a publicly available ChIP-seq dataset (32), we identified the FOXF1-binding region in the –267/+0 bp *Ifitm3* promoter (Figure 6H). To functionally validate this FOXF1-DNA interaction, we cloned the *Ifitm3* promoter region into a pGL4.23 luciferase reporter plasmid. In cotransfection experiments, the Foxf1 expression vector increased luciferase activity, whereas the empty luciferase vector had no effect (Figure 6I). In colocalization studies using both human and mouse lung tissue sections, we demonstrated that IFITM3 protein colocalized with capEPCs (Supplemental Figure 6, A and B). In IPF human and bleomycin-treated mouse lungs, the IFITM3 signal intensity and the capEPC abundance were markedly reduced (Supplemental Figure 6, A and B). Thus, IFITM3 is expressed in capEPCs and FOXF1 directly activates transcription of *Ifitm3*, indicating that the *Ifitm3* gene is a direct transcriptional target of FOXF1.

### IFITM3 improves EC functions but inhibits fibroblast activation.

Next, we examined the effects of IFITM3 on lung ECs and fibroblasts in vitro. Mouse MFLM-91U cells were transfected with CMV-Ifitm3 plasmid to overexpress IFITM3 (Figure 7A). Conditioned medium from Ifitm3-overexpressing ECs increased endothelial tube formation in an in vitro assay (Figure 7B). In addition, treatment of human pulmonary artery endothelial cells (HPAECs) with recombinant IFITM3 (rIFITM3) protein significantly improved tube formation in vitro and increased the number of HPAECs in culture (Figure 7, C and D). These results were verified using human pulmonary microvascular endothelial cells (HPMECs), demonstrating that treatment with rIFITM3 increased angiogenesis and numbers of HPMECs in vitro (Supplemental Figure 6, C–E). In contrast to the activating effect of IFITM3 on ECs, the rIFITM3 treatment decreased proliferation and migration of CCD-19Lu fibroblasts (Figure 7, E and F) and inhibited profibrotic *COL3A1*, *ACTA2*, and *VIM* mRNAs in lung fibroblasts (Figure 7G). Thus, IFITM3 increased ECs’ pro-angiogenic properties but inhibited fibroblast activation in vitro.

### IFITM3 is exported through an exosome-mediated pathway.

Since IFITM3 lacks a signaling peptide for conventional protein secretion (33) (Supplemental Figure 6, F and G), and its export occurs through an exosome-mediated pathway (34), we tested the function of IFITM3 in exosomes. We generated an HPAEC cell line that expressed exogenous IFITM3 protein fused with an 11–amino acid HiBiT tag that possessed bioluminescence activity. Furthermore, the IFITM3-HiBiT fusion protein was expressed under control of doxycycline (Dox). Addition of Dox led to overexpression of exogenous IFITM3, which was detected by luminescence (Figure 8A). Endogenous IFITM3 in this cell line was inhibited by FOXF1-targeting siRNA (Figure 8A). The HiBiT tag enabled the detection of the IFITM3-HiBiT fusion protein in exosomes using the Nano-Glo HiBiT Extracellular Detection System. Exosomes were isolated from the conditioned medium of parental HPAECs or siFOXF1-transfected HPAECs in the presence or absence of Dox. Dynamic light scattering analysis confirmed that the isolated exosomes had an expected size distribution with a major peak intensity ranging between 94.50 ± 11.37 nm and 111.47 ± 21.88 nm, with no significant differences observed between groups (Figure 8B and Supplemental Table 1). Bioluminescence analysis of purified exosomes demonstrated increased bioluminescence activity in Dox-treated samples compared with exosomes from parental cells (control) or exosomes from cells without Dox, consistent with the presence of exogenous HiBiT-IFITM3 protein in exosomes after Dox treatment (Figure 8C).

### Treatment with IFITM3-containing exosomes improves EC functions but inhibits fibroblast activation.

To test the functions of IFITM3 in exosomes, we purified exosomes from parental HPAECs (Exo Control) or siFOXF1-transfected HPAECs cultured without Dox (IFITM3^lo^ exosomes) or with Dox (IFITM3^hi^ exosomes). Endothelial cells were treated with these exosomes to test their effects in vitro. The IFITM3^lo^ exosomes decreased tube formation in ECs compared with control exosomes as evidenced by reduced length of EC sprouts (Figure 8D). The tube formation in ECs was restored when the cells were treated with IFITM3^hi^ exosomes (Figure 8D). We next tested the impact of IFITM3-containing exosomes on fibroblast activation. Consistent with published studies, addition of TGF-β1 to culture media increased human CCD-19Lu fibroblast proliferation as previously shown (35). IFITM3^lo^ exosomes further increased TGF-β1–induced fibroblast proliferation at 48 hours after treatment. In contrast, IFITM3^hi ^exosomes reduced fibroblast proliferation at both 24 and 48 hours after TGF-β1 treatment (Figure 8E). Furthermore, IFITM3^hi^ exosomes decreased profibrotic *FN1*, *VIM*, and *COL3A1* mRNAs in TGF-β1–activated fibroblasts (Figure 8F). Altogether, IFITM3-containing exosomes improved EC tube formation but inhibited TGF-β1–induced fibroblast activation in vitro.

### Treatment with IFITM3-containing exosomes improves survival and lung functions in mouse model of lung fibrosis.

To evaluate the therapeutic effects of IFITM3-containing exosomes in lung fibrosis, mice were intratracheally injected with IFITM3^hi^ exosomes or control IFITM3^lo^ exosomes on days 8 and 11 after 2 bleomycin administrations (Figure 9A). While mice treated with control exosomes exhibited continuous body weight loss throughout the study period, mice treated with IFITM3^hi^ exosomes showed improved recovery of body weights (Figure 9A). Survival rates were also improved in the IFITM3^hi^ exosome–treated group compared with the control group (Figure 9B). Luminescence analysis of lung tissues revealed higher HiBiT activity in the IFITM3^hi^ exosome–treated group (Figure 9C), which was consistent with increased immunostaining of lung tissue sections using anti-HiBiT antibody and increased IFITM3 protein levels (Supplemental Figure 7A). Treatment with IFITM3^hi^ exosomes restored peripheral blood oxygen saturation after bleomycin injury (Figure 9D). Lung mechanics measured by flexiVent showed significant improvement in the IFITM3^hi^ exosome–treated group, including decreased elastance and resistance, and increased compliance and inspiratory capacity (Figure 9, E–H). Furthermore, treatment with IFITM3^hi^ exosomes decreased lung fibrosis as shown by reduced hydroxyproline levels (Figure 9I), histological staining of lung tissue using H&E, Masson’s trichrome staining, and Sirius red/fast green (Figure 9J), and PDGFRA^+^ cells (Supplemental Figure 7B), Ashcroft score (Figure 9K), and qRT-PCR for fibrosis-associated mRNAs (Figure 9L). Finally, treatment with IFITM3^hi^ exosomes increased abundance of lung pro-SPC^+^ epithelial cells (Supplemental Figure 7B). Altogether, intratracheal delivery of IFITM3-containing exosomes improved survival, body weight, and lung function and decreased PF in bleomycin-injured mice.

## Discussion

Endothelial progenitor cells (EPCs) are defined by their proliferative and angiogenic potential, traditionally characterized as CD34^+^ mononuclear cells derived from the bone marrow that express endothelial markers and progenitor-associated antigens while lacking hematopoietic markers (36). In fetal and neonatal lungs, a FOXF1^+^KIT^+^ pulmonary resident EPC population has been identified as critical for alveolarization and postnatal angiogenesis in the developing alveoli (22–24). Published studies showed that adoptive transfer of EPCs is effective in murine models of neonatal lung diseases, including bronchopulmonary dysplasia (BPD) and alveolar capillary dysplasia, preventing alveolar simplification and improving angiogenesis (22, 24, 37). However, to date, there has been no definitive study characterizing a FOXF1^+^KIT^+^ resident EPC population in adult lungs or showing their therapeutic potential in adult respiratory diseases. In this study, we provide what we believe to be the first evidence that a FOXF1^+^KIT^+^ (capEPC) population exists in adult lungs across species. These cells are present predominantly among the general capillary ECs. We used cross-species gene expression and pathway enrichment analyses to show that gene expression signatures of capEPCs in both mouse and human lungs are enriched for signaling pathways related to extracellular matrix organization, neovascularization, cadherin signaling, and KIT receptor–mediated transcriptional regulation, implicating the adult capEPCs in tissue remodeling and vascular regeneration. Notably, we observed a reduction in the number of capEPCs in human and mouse fibrotic lungs, suggesting that the loss of these cells can play a role in PF pathogenesis. Interestingly, based on single-cell RNA sequencing, capEPCs are distinct from ABCG2^+^ EPCs identified previously (16) (Supplemental Figure 8, A and B).

EPCs are thought to participate in vascular repair through two primary mechanisms: direct incorporation into the endothelium and paracrine signaling, including the release of exosomes and other bioactive factors (12, 38, 39). In our study, we demonstrated that transplantation of adult lung capEPCs attenuates PF. Although only a small fraction of transplanted cells remained in recipient lungs, therapeutic benefit was still observed, suggesting additional paracrine effects. This observation aligns with prior studies showing that neonatal lung FOXF1^+^KIT^+^ cells integrate into alveolar microvasculature and reverse alveolar simplification in a genetic model of alveolar capillary dysplasia (24). Donor EPCs improved lung repair in neonatal BPD models and required interactions with alveolar macrophages that enhance EPC retention and efficacy (37). Our findings suggest that therapeutic efficacy of donor capEPCs does not require widespread engraftment but can be driven by targeted niche integration and paracrine mechanisms.

In the present study, we found that capEPCs secrete IFITM3-containing exosomes, which may mediate antifibrotic effects. IFITM3 functions primarily through modulation of membrane biophysics and intracellular trafficking rather than via classical ligand-receptor interactions (40, 41). While IFITM3 is well known for its antiviral properties, to our knowledge, our study is the first to demonstrate its ability to enhance EC proliferation and tube formation, as well as to inhibit fibroblast proliferation, extracellular matrix production, and migration. Beyond its antiviral properties, IFITM3 also modulates innate immunity by binding to IRF3 and promoting its degradation through autophagy (42). This selectively dampens type I interferon signaling mediated by RIG-I–like receptors, without affecting other pathways such as TLR3 or IFN-γ–induced IRF1 activation (42). IFITM3 interacts with the tetraspanin CD151 at endolysosomal membranes, where CD151 anchors IFITM3 to the VCP/p97 complex to restrict vesicular trafficking (43, 44). Disruption of this interaction enhances endothelial inflammation and may contribute to PF (43, 44).

IFITM3 is a transmembrane protein and is not secreted as a soluble protein through the conventional secretory pathway. We and others have demonstrated that both human and mouse IFITM3 lacks a signal peptide required for classical protein secretion (33). However, since IFITM3 is a transmembrane protein localized to endosomal and membrane compartments, previous studies have shown that IFITM3 can be incorporated into extracellular vesicles (33, 34). To examine the importance of EVs in extracellular transport of IFITM3, we generated an IFITM3-HiBiT–expressing endothelial reporter cell line and detected HiBiT signal in isolated extracellular vesicles, verifying that IFITM3-HiBiT is released through an EV-associated mechanism. Taken together, the lack of a signal peptide, the membrane topology of IFITM3, and our reporter HiBiT-based EV detection collectively support that IFITM3 is transported via extracellular vesicles to mediate its antifibrotic and angiogenic effects.

Exosomes have increasingly gained attention as a novel therapeutic strategy for PF. Notably, lung spheroid cell–derived exosomes have shown superior efficacy compared with mesenchymal stromal cell–derived (MSC-derived) exosomes in reversing fibrosis and restoring alveolar structure and function (45). MSC-derived exosomal microRNA-218 attenuated bleomycin-induced PF by inhibiting endothelial-mesenchymal transition (46). EPC-derived exosomes reduce endothelial permeability and promote vascular repair in lipopolysaccharide-induced acute lung injury models, demonstrating their therapeutic relevance in correcting endothelial dysfunction (47, 48). Beyond their intrinsic bioactivity, exosomes offer unique advantages as drug delivery vehicles due to their superior biocompatibility, low immunogenicity, and innate tissue-targeting capacity. Their ability to carry both hydrophilic and lipophilic agents, such as pirfenidone, makes them well suited for PF therapy (49). While challenges such as large-scale production and regulatory standardization remain (50), exosomes hold strong potential as both therapeutic agents and delivery platforms in PF treatment. Delivery of IFITM3-enriched exosomes may represent promising therapeutic strategies for human PF.

Our data support a model in which the capEPC-FOXF1-IFITM3 mechanism enhances endothelial regenerative capacity during lung fibrogenesis. IFITM3 has been reported to regulate membrane dynamics, endosomal trafficking, and innate immune signaling pathways, which may influence angiogenic signaling and cellular activation states. IFITM3-high exosomes promote endothelial tube formation and increase the number of ECs in vitro, and increase vascular density and EC proliferation in vivo, while simultaneously suppressing fibroblast activation and collagen deposition in vitro and in vivo. In summary, we provide evidence that capEPCs exist in the adult lung and that their numbers are decreased in mouse and human PF. Transplantation of donor capEPCs attenuates lung fibrosis in a mouse bleomycin model through at least two mechanisms: direct engraftment into the lung tissue and secretion of IFITM3-containing exosomes. Donor capEPCs hold promise for future therapies in PF.

## Methods

### Sex as a biological variable.

In preclinical in vivo studies, both female and male mice were used, and experimental groups were balanced by sex. Human lung samples were obtained from both male and female donors.

### Bleomycin-induced fibrosis animal models.

C57BL/6 and Foxf1-GFP knockin mice (8–12 weeks old, both sexes) were used (24). Lung fibrosis was induced by intratracheal administration of bleomycin sulfate (2 mg/kg; catalog B5507, Sigma-Aldrich; in sterile PBS) on days 0 and 7. Mice were monitored for survival, body weight, and lung function.

### Flow cytometry and sorting.

Flow cytometry on mouse lung tissue was performed following established protocols (30), using antibodies listed in Supplemental Table 2. Stained cells were analyzed using the Aurora flow cytometer (Cytek Biosciences) and sorted using the FACSAria II (BD Biosciences), SH800 (Sony), and MA900 (Sony) sorters. Data were acquired using FlowJo v10.8.0 and FACSDiva v9.0. Magnetic-activated cell sorting (MACS) was performed to isolate CD45^–^CD31^+^CD117^+^ cells using magnetic beads (Miltenyi Biotec). Sorted cells were cultured in EGM-2/EBM-2 medium (catalog CC-3202, Lonza) on Attachment Factor–coated plates (catalog 4Z0-201, Cell Systems) at 37°C with 5% CO_2_.

### Transplantation of donor capEPCs.

Donor capEPCs were FACS-sorted from the lungs of adult Foxf1-GFP;CAG-tdTomato mice (24). Lung ECs (CD45^–^CD31^+^CD117^+^tdTomato^+^) were subdivided into GFP^+^ (capEPC) or GFP^–^ (control Foxf1^–^Kit^+^ EC) subpopulations. Recipient C57BL/6 mice were subjected to bleomycin-induced lung fibrosis via intratracheal administration of bleomycin sulfate (2 mg/kg in sterile PBS) on days 0 and 7. On day 8 after bleomycin, the donor capEPCs or control cells were intravenously injected into the recipient mice. The other control was the PBS-treated mice (mock control). Survival, body weight, and lung function were monitored, and therapeutic outcomes were evaluated on day 21.

### Intratracheal delivery of exosomes.

Bleomycin-treated mice received intratracheal injections of IFITM3-containing or control exosomes (20 μg in 50 μL PBS) on days 8 and 11. Mice were monitored for survival, body weight, and lung function, with experimental endpoints assessed on day 15.

### Construction of the mouse Ifitm3 promoter region and luciferase assay.

The mouse *Ifitm3* promoter region (–267 bp to 0 bp) was PCR amplified from CD1 genomic DNA using the primers listed in Supplemental Table 3 and cloned into the KpnI site of pGL4.23[luc2/minP] (catalog E8411, Promega). MFLM-91U cells were cotransfected with this construct, CMV-Foxf1, or empty vector as described previously (23), and Renilla plasmid control vector (catalog E6931, Promega). Luciferase activity was quantified using a dual-luciferase assay.

### Construction of pTetOne-HiBiT-IFITM3 and Nano-Glo HiBiT extracellular detection assay.

The IFITM3 coding sequence was amplified from the pCMV-HA-hIFITM3 plasmid (catalog 58397, Addgene), fused in-frame with the HiBiT tag, and cloned into the pTetOne vector from the Tet-One Inducible Expression System (catalog 634301, Takara Bio USA Inc.) using In-Fusion Snap Assembly Master Mix (catalog 638947, Takara Bio) according to the manufacturer’s instructions. Primers used for amplification are listed in Supplemental Table 3.

For HiBiT detection (catalog N3030, Promega), 100 μL of conditioned medium or lysate was mixed with detection reagent and incubated at room temperature for 10 minutes. Luminescence was measured using a Cytation 1 plate reader (BioTek).

### scRNA-seq analysis.

Human lung endothelial scRNA-seq data (GEO GSE227136) (25) were reanalyzed using Seurat v4.4.0 in R v4.3.1. The dataset includes 114 individuals (48 controls and 66 with lung disease). To capture spatial heterogeneity, fibrotic samples were separated into “more fibrotic” and “less fibrotic” regions, resulting in 2 libraries per subject. A pre-processed Seurat object combining 24 flow cell batches (GSE227136_ILD_endothelial_Seurat.rds.gz) was used for downstream analysis. Highly variable genes (*n* = 4,000) were identified using the variance-stabilizing transformation method. SCTransform normalization and principal component analysis (PCA; top 50 PCs) were performed, and 44 PCs were selected based on variance explained. Datasets were first integrated using reciprocal PCA with 3,000 anchors, followed by batch correction using Harmony to mitigate flow cell– and donor-associated effects (51). UMAP was generated from the Harmony-corrected PCA space (dims = 1:44), and clustering was performed using the shared nearest neighbor (SNN) graph at resolution 0.2. Endothelial clusters were annotated using canonical markers for general capillary (CAP1), aerocyte (CAP2), arterial (AEC), venous (VEC), and systemic venous endothelial cell (SVEC) subtypes. AECs and VECs were further reclustered to identify activated states. FOXF1 and KIT were co-loaded on PC_44. Expression of key genes was visualized using UMAP, FeaturePlot, and heatmaps. Differentially expressed genes (DEGs) were identified using FindAllMarkers (Satija Lab; https://satijalab.org/seurat/reference/findallmarkers) (log_2 _fold change [log_2_FC] ≥ 0.25, adjusted *P* ≤ 0.05) and subjected to pathway analysis via Enrichr (Ma’ayan Lab; https://maayanlab.cloud/Enrichr/) using Fisher’s test and activation *z* score. All plots were generated using Seurat and ggplot2 (https://ggplot2.tidyverse.org/).

Raw mouse endothelial scRNA-seq data (GSE264151 and GSE264162) (28) were processed in Seurat v4.4.0. Cells with ≥400 unique molecular identifiers (UMIs), ≥200 genes, log_10_ genes per UMI > 0.8, and <20% mitochondrial content were retained. Genes detected in fewer than 10 cells were excluded. Endothelial cells were selected based on marker expression and analyzed separately. Each dataset was SCTransformed and integrated via reciprocal PCA using 3,000 anchor features, followed by Harmony-based batch correction. PCA and UMAP (dims = 1:40) were used for dimensionality reduction, followed by SNN-based clustering (resolution 0.1–1.4). EC subtypes (CAP1, CAP2, AEC, VEC) were annotated and subclustered for activation state analysis. Gene expression was visualized using UMAPs, dot plots, and heatmaps. DEGs were identified with FindAllMarkers (log_2_FC ≥ 0.25). Integrated Seurat objects were used for comparative analysis between species.

### Differential gene expression between capEPCs and Foxf1^–^Kit^+^ ECs.

Single-cell RNA-seq data from adult wild-type mouse lung ECs were analyzed using Seurat v4.4.0 in R. Kit^+^ ECs were stratified into Foxf1^+^ (capEPCs) and Foxf1^–^ populations. DEGs were identified using FindAllMarkers (log_2_FC > 1, Bonferroni-adjusted *P* < 0.05) and visualized with ggplot2.

### Cell culture, RNA interference, and qRT-PCR.

Human pulmonary artery endothelial cells (HPAECs; catalog CC-2530, Lonza) and human pulmonary microvascular endothelial cells (HPMECs; catalog 3000, ScienCell Research Laboratories) were cultured in ECM medium (catalog 1001, ScienCell Research Laboratories). Mouse endothelial MFLM-91U cells, derived from murine fetal lung mesenchyme (catalog AMFLM-91U, Seven Hills), and NIH 3T3 fibroblasts, derived from embryonic mouse fibroblasts (catalog CRL-1658, ATCC), were maintained in DMEM (catalog 11965092, Thermo Fisher Scientific) supplemented with 10% fetal bovine serum (catalog A5209502, Thermo Fisher Scientific). Human lung fibroblasts CCD-19Lu, derived from the lung of a 20-year-old female (catalog CCL-210, ATCC), were cultured in Eagle’s Minimum Essential Medium (catalog 30-2003, ATCC).

ON-TARGETplus siRNAs targeting FOXF1 (mouse: catalog L-043272-01-0010; human: catalog L-009628-00-0010; Horizon Discovery) were transfected into MFLM-91U cells, MACS-sorted Kit^+ ^ECs, and HPAECs using DharmaFECT 1 transfection reagent (catalog T-2001-02, Dharmacon), following the manufacturer’s instructions. A non-targeting siRNA pool (catalog D-001810-01-10, Horizon Discovery) was used as control. For rescue experiments, HPAECs were cotransfected with siFOXF1 and the pTetOne-HiBiT-IFITM3 plasmid using a TransIT-X2 Dynamic Delivery System (catalog MIR 6004, Mirus Bio), followed by doxycycline treatment for 24 hours.

RNA was extracted from cells using the Quick-RNA Microprep Kit (catalog R1051, Zymo Research). cDNA synthesis was performed using either the High-Capacity cDNA Reverse Transcription Kit (catalog 4368814, Thermo Fisher Scientific) or the iScript cDNA Synthesis Kit (catalog 1708891, Bio-Rad), according to manufacturers’ protocols. Quantitative gene expression analysis was conducted by TaqMan-based qRT-PCR using probes listed in Supplemental Table 4.

### Tube formation assay.

MFLM-91U cells or HPAECs were seeded on Matrigel-coated plates and incubated for 12–24 hours. Cells were stained with Calcein AM (2 μg/mL; catalog C1430, Thermo Fisher Scientific) for 15 minutes at 37°C. Fluorescence images were acquired using the EVOS FL Auto 2 Cell Imaging System (Thermo Fisher Scientific) and analyzed with AngioTool software v0.6a (52).

### Migration assay.

CCD-19Lu fibroblasts were seeded into 2-well culture inserts (catalog 80209, ibidi GmbH) and allowed to adhere overnight. Inserts were removed to create a cell-free gap. Cells were treated with recombinant human IFITM3 protein (100 ng/mL; catalog 12431-H04H, Sino Biological Inc.) or left untreated for 30 hours, stained with Calcein AM, and imaged. Migration was quantified using TScratch software (https://github.com/cselab/TScratch; commit ID 27c6e9a) by calculation of the percentage of the remaining open area.

### Exosome isolation and characterization.

Exosomes were isolated from HPAEC-conditioned medium using the Total Exosome Isolation Reagent (catalog 4478360, Thermo Fisher Scientific) and resuspended in Dulbecco’s phosphate-buffered saline. Size and distribution were measured by dynamic light scattering using a Zetasizer Ultra (Malvern Instruments). Concentration was determined using a BCA Protein Assay Kit (catalog 23227, Thermo Fisher Scientific). IFITM3 levels were measured by Nano-Glo HiBiT detection (Promega).

### Coculture of exosomes with fibroblasts.

CCD-19Lu fibroblasts were seeded at 1 × 10^4^ cells per well in 48-well plates and treated with TGF-β1 (10 ng/mL; catalog 10804-H08H1, Sino Biological Inc.) and exosomes (2 μg/mL) from HPAECs transfected with siFOXF1 and pTetOne-HiBiT-IFITM3, with or without doxycycline, for 24–48 hours. Proliferation was assessed by cell counts. For extracellular matrix gene analysis, cells were harvested at 48 hours for RNA extraction and analyzed by TaqMan-based qRT-PCR using probes listed in Supplemental Table 4.

### Immunostaining and collagen content.

Lung tissues were processed for histology and immunofluorescence staining. H&E staining was performed on paraffin sections. Both paraffin- and OCT compound–embedded frozen sections were used for immunofluorescence following standard protocols. Primary antibodies are listed in Supplemental Table 5. Alexa Fluor–conjugated secondary antibodies (488, 594, 647; Thermo Fisher Scientific) were used, and nuclei were counterstained with Hoechst 33342 (catalog H1399, Thermo Fisher Scientific). Fluorescent images were acquired using the EVOS FL Auto 2 Cell Imaging System (Thermo Fisher Scientific) and analyzed with EVOS software. Collagen content was assessed using the hydroxyproline assay (catalog STA-675, Cell Biolabs Inc.), Sirius red/fast green staining (catalog 9046, Chondrex), and Masson’s trichrome staining (catalog 25088, Poly Scientific), according to manufacturers’ protocols. Quantification was performed using ImageJ (NIH).

### Respiratory mechanics and arterial oxygenation.

Respiratory system mechanics and arterial oxygenation were assessed on day 15 or 21 after bleomycin administration. Arterial oxygen saturation was measured noninvasively using the MouseOx Plus system (STARR Life Sciences) under ketamine/xylazine anesthesia. The photodiode sensor was placed on the shaved neck, and data were recorded for 3 minutes using MouseOx software v1.6.X. Respiratory mechanics was assessed under sodium pentobarbital anesthesia using the flexiVent system (SCIREQ). Parameters including airway resistance and compliance were analyzed using flexiWare v8.1.

### Statistics.

Statistical analyses were performed using GraphPad Prism version 9.0 for Windows (GraphPad Software) and R software version 4.5.2. Differences between 2 groups were assessed using unpaired 2-tailed Student’s *t* test or Wilcoxon’s rank-sum test (Mann-Whitney *U* test), as appropriate. Comparisons among multiple groups were performed using 1-way ANOVA followed by Fisher’s least significant difference post hoc test, or by Kruskal-Wallis test followed by pairwise Wilcoxon’s rank-sum tests with Benjamini-Hochberg correction for multiple comparisons, as indicated in the figure legends. Two-way ANOVA with Greenhouse-Geisser correction was used for analyses involving multiple groups and time points. For integrated analyses of multiple datasets, linear models with dataset covariates were applied as indicated. Data are presented as mean ± SD, and *P* < 0.05 was considered statistically significant. All experiments were performed independently at least 3 times, with similar results.

### Study approval.

Deidentified lung tissue specimens from healthy controls and patients with idiopathic pulmonary fibrosis were obtained from the tissue repositories at the University of Cincinnati Medical Center (Cincinnati, Ohio, USA) and St. Joseph’s Hospital in Arizona. The use of deidentified human lung tissue was approved by the Institutional Review Board at the University of Arizona. All animal procedures were conducted in accordance with protocols approved by the Institutional Animal Care and Use Committee at the University of Arizona (protocol 2023-1128). The Foxf1-GFP knockin and Foxf1-GFP;CAG-tdTomato mouse lines used in this study have been described previously (23).

### Data availability.

All data associated with this study are present in the paper. Single-cell RNA sequencing datasets from donor and PF lungs were retrieved from the NCBI’s Gene Expression Omnibus (GEO) database (accession number GSE227136, https://www.ncbi.nlm.nih.gov/geo/query/acc.cgi?acc=GSE227136). Single-cell RNA sequencing from bleomycin-treated and control mouse lungs was retrieved from the GEO database (accession number GSE264151, https://www.ncbi.nlm.nih.gov/geo/query/acc.cgi?acc=GSE264151; and GSE264162, https://www.ncbi.nlm.nih.gov/geo/query/acc.cgi?acc=GSE264162). The single-cell RNA sequencing dataset generated in this study from adult wild-type mouse lungs was deposited in the GEO database (accession number GSE341505, https://www.ncbi.nlm.nih.gov/geo/query/acc.cgi?acc=GSE341505). The ChIP-seq dataset from mouse lungs was retrieved from the GEO database (accession number GSE77951, https://www.ncbi.nlm.nih.gov/geo/query/acc.cgi?acc=GSE77951). Values for all data points shown in graphs and values underlying reported means are provided in the Supporting Data Values file.

## Author contributions

YWL and TVK conceived and designed the study. YWL, JD, WG, ZD, XX, GV, and JK performed the experiments. YWL, NEB, and EL performed the scRNA-seq, RNA-seq, and ChIP-seq bioinformatics analysis. YWL and TVK analyzed data. YWL, VVK, and TVK interpreted the data. JD, AS, KSK, RMB, and VVK provided critical samples and intellectual discussions. YWL and TVK wrote the paper. All authors discussed the data. TVK approved the submission of the manuscript.

## Conflict of interest

The authors have declared that no conflict of interest exists.

## Funding support

This work is the result of NIH funding, in whole or in part, and is subject to the NIH Public Access Policy. Through acceptance of this federal funding, the NIH has been given a right to make the work publicly available in PubMed Central.

- National Institutes of Health (NIH) grants R01 HL132849 to TVK, R01 HL158659 to TVK, R01 HL141174 to VVK, R01 HL149631 to VVK, and R01 HL152973 to TVK and VVK.
- Arizona Biomedical Research Centre funding RFGA2024-022-001 to YWL.

## Supplementary Material

Supplemental data

Supporting data values

## Figures and Tables

**Figure 1:**
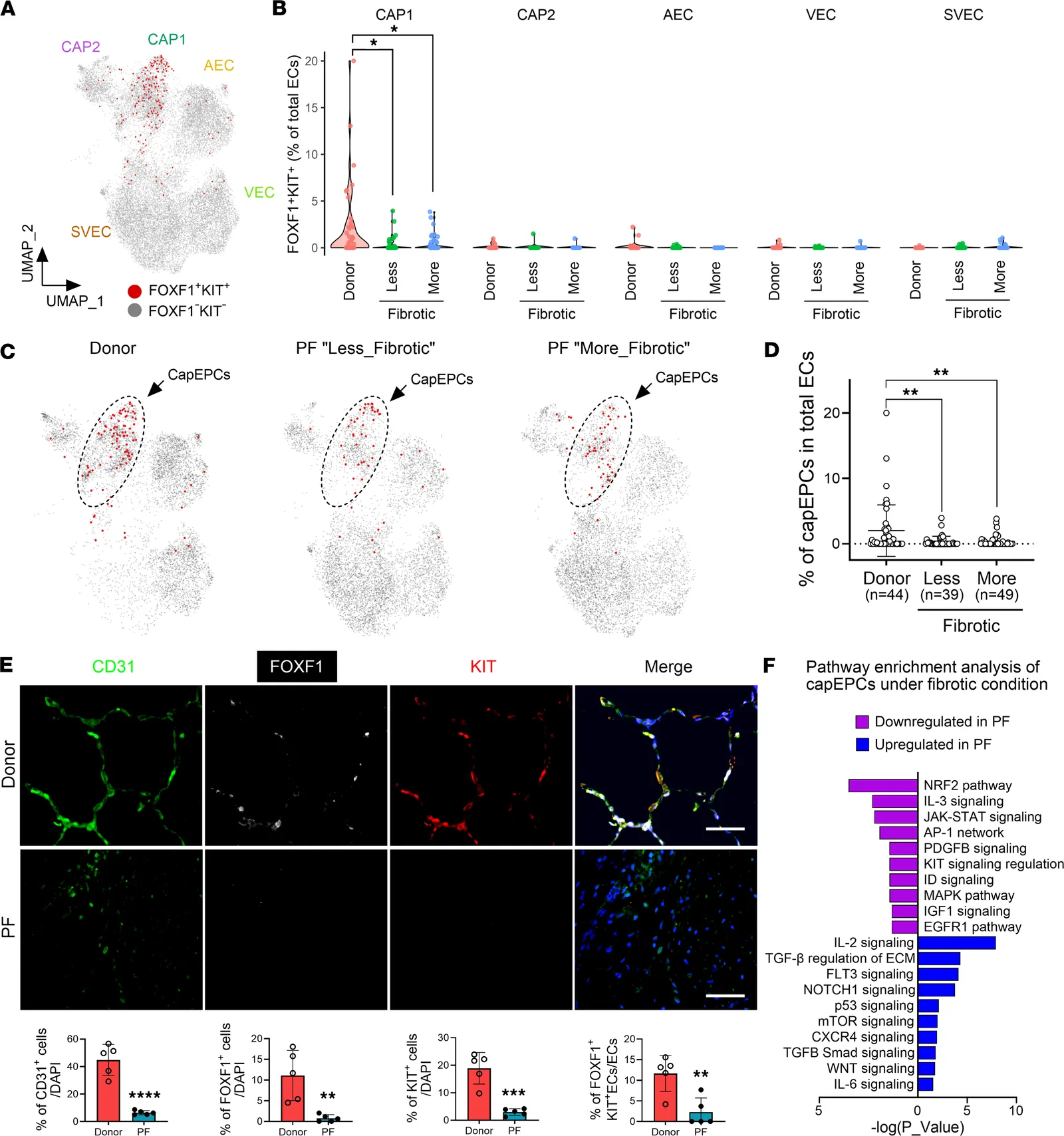
Lung capEPCs localize within CAP1 and are decreased in fibrotic lungs of PF patients. (**A**) UMAP visualization of EC subclusters (*n* = 37,752 cells) from donor and PF lungs (GSE227136), highlighting KIT^+^FOXF1^+^ cells (capEPCs) in red. (**B**) Quantification of FOXF1^+^KIT^+^ capEPCs across endothelial subtypes in donor, less fibrotic PF, and more fibrotic PF lungs. Data shown as the percentage of FOXF1^+^KIT^+^ cells among total ECs, calculated separately for each sample. Each dot represents an individual donor or PF patient sample. Horizontal lines indicate mean. Statistical significance was assessed using the Kruskal-Wallis test followed by pairwise Wilcoxon’s rank-sum tests with Benjamini-Hochberg correction. (**C**) Reduced abundance of capEPCs in fibrotic lungs. Left: UMAP visualization showing capEPCs (red dots) in donor, less fibrotic PF, and more fibrotic PF lungs. Dashed outlines indicate capEPC-enriched region. Right: Percentage of capEPCs among total ECs: donor (*n* = 44), less fibrotic PF (*n* = 39), and more fibrotic PF (*n* = 49). (**D**) Top: Representative immunofluorescence images of donor and PF lung sections stained for CD31 (green), FOXF1 (white), KIT (red), and DAPI (blue). Bottom: CD31^+^, FOXF1^+^, and KIT^+^ cells are shown as percentages of total DAPI^+^ nuclei, and capEPCs as a percentage of total CD31^+^ ECs, calculated per lung sample. PF lungs showed a significant reduction in capEPCs compared with donor lungs. *n* = 5 lungs/group. Scale bars: 100 μm. (**E**) Pathway enrichment analysis of genes differentially expressed in capEPCs from donor and PF lungs. Pathways downregulated or upregulated in PF capEPCs. Data presented as mean ± SD. *P < 0.05, **P < 0.01, ***P < 0.001, ****P < 0.0001. One-way ANOVA, Fisher’s least significant difference (LSD).

**Figure 2:**
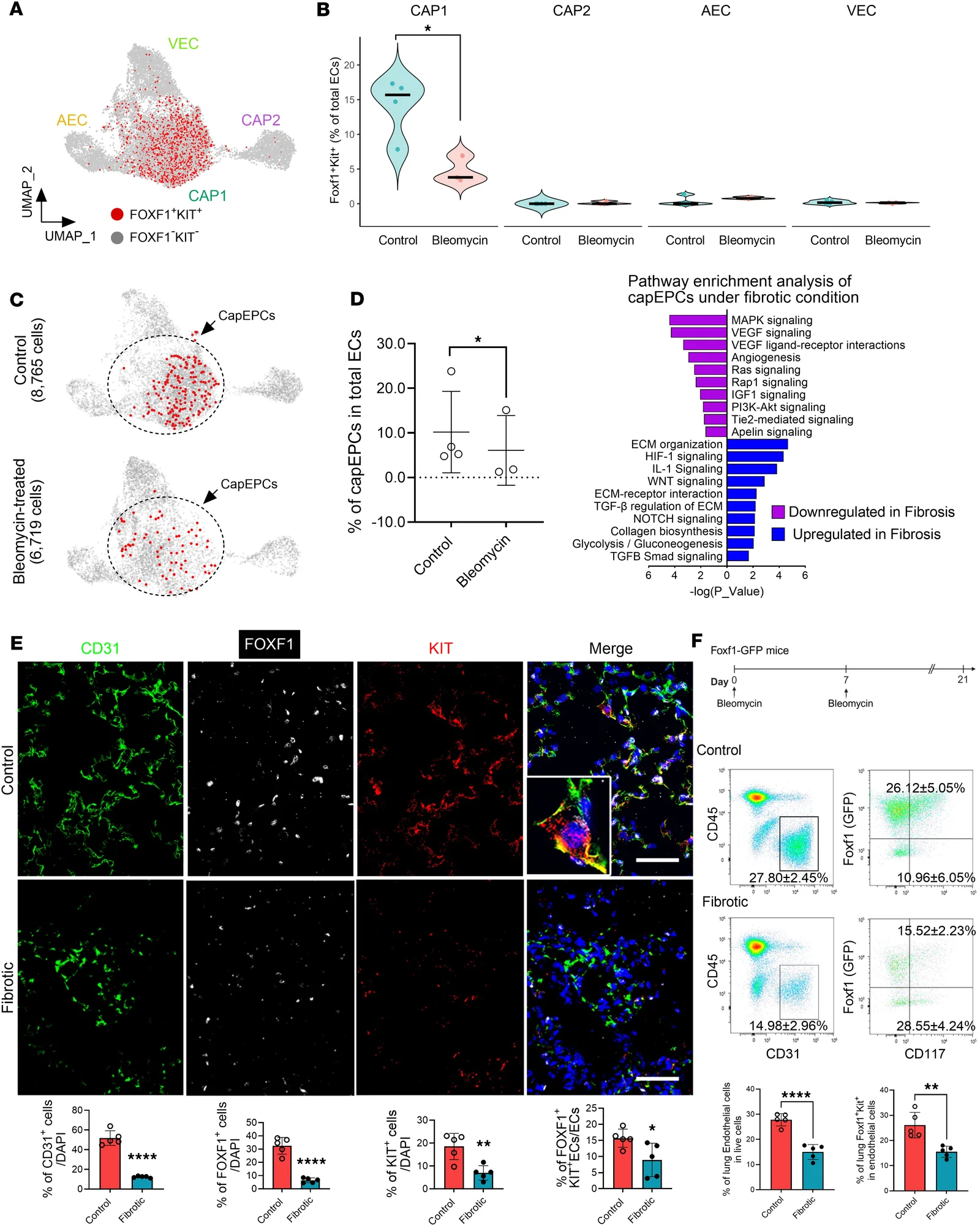
Mouse lung capEPCs localize within CAP1 and are decreased in lungs of bleomycin-treated mice. (**A**) UMAP visualization of lung EC subclusters from control and bleomycin-treated mouse lungs (GSE264151 and GSE264162), highlighting capEPCs (FOXF1^+^KIT^+^) in red. (**B**) Quantification of FOXF1^+^KIT^+^ capEPCs across endothelial subtypes in control and bleomycin-treated lungs. Data are shown as the percentage of FOXF1^+^KIT^+^ cells among total ECs within each subtype, calculated separately for each mouse lung. Each dot represents an individual mouse. Horizontal lines indicate the mean. Statistical significance was assessed using the Wilcoxon’s rank-sum test. (**C**) Reduced abundance of capEPCs following bleomycin injury. Left: UMAP visualization of FOXF1^+^KIT^+^ capEPCs (red dots) in control and bleomycin-treated lungs. Dashed outlines indicate the capEPC-enriched region. Right: Percentage of capEPCs among total ECs, calculated per mouse lung (control, *n* = 4; bleomycin, *n* = 3). Statistical significance was assessed using a linear model with dataset covariate adjustment. (**D**) Pathway enrichment analysis of capEPCs under fibrotic conditions, showing signaling pathways up- or downregulated following bleomycin treatment. (**E**) Top: Immunofluorescent staining of lung sections from control (*n* = 6) and bleomycin-treated mice (*n* = 8) for CD31 (green), FOXF1 (white), KIT (red), and DAPI (blue). Bottom: Quantification of CD31^+^, FOXF1^+^, and KIT^+^ cells expressed as a percentage of total DAPI^+^ nuclei, and FOXF1^+^KIT^+^ capEPCs expressed as a percentage of total CD31^+^ ECs, calculated per lung. Representative images from independent lungs are shown. Scale bars: 100 μm. (**F**) Flow cytometry analysis using Foxf1-GFP reporter mice (experimental design shown above) shows decreased percentage of capEPCs (CD117^+^Foxf1^+^ ECs) in bleomycin-treated lungs (*n* = 5 mice per group). Data are presented as mean ± SD. *P < 0.05, **P < 0.01, ****P < 0.0001. For **E** and **F**, unpaired 2-tailed *t* tests were used.

**Figure 3:**
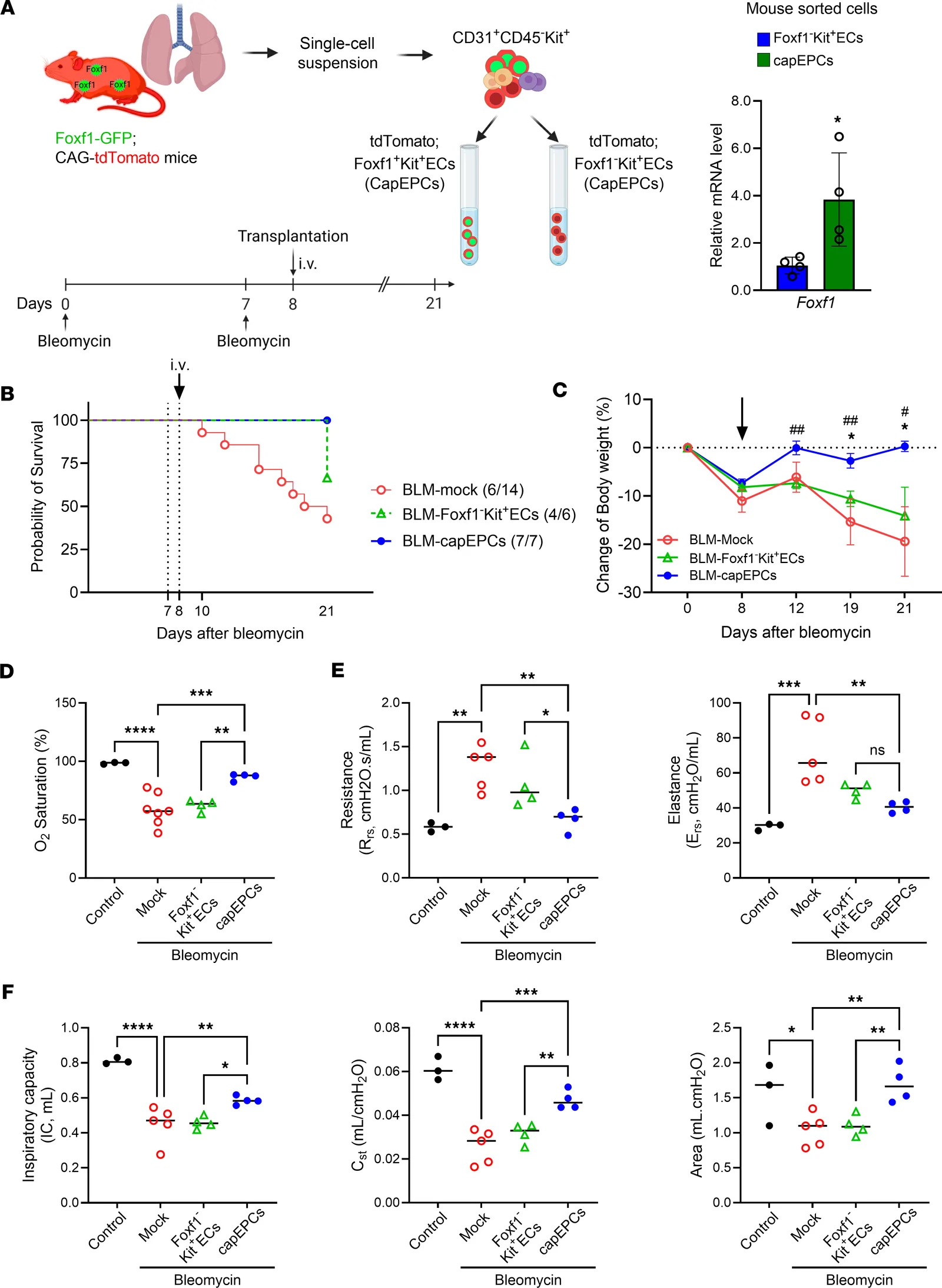
Transplantation of capEPCs improves survival and lung function in bleomycin-injured mice. (**A**) Top: Schematic of the FACS sorting strategy to isolate donor capEPCs (Foxf1^+^Kit^+^) and control Foxf1^–^Kit^+^ ECs from Foxf1-GFP;CAG-tdTomato reporter mice. Bottom: Experimental timeline. Right: qRT-PCR confirmed higher *Foxf1* mRNA in capEPCs (*n* = 4). (**B**) Transplantation of capEPCs improved survival of bleomycin-injured mice. Numbers in parentheses indicate surviving mice/total mice per group. (**C**) Body weight recovery after bleomycin injury was improved in mice treated with capEPCs compared with mock-treated mice (BLM-mock) or mice treated with Foxf1^–^Kit^+^ ECs (BLM-Foxf1^–^Kit^+^). *Comparison with BLM-mock; ^#^comparison with BLM-Foxf1^–^Kit^+^ ECs. (**D**) Arterial oxygen saturation on day 21 was increased after capEPC transplantation (control, *n* = 3; BLM-mock, *n* = 7; BLM-Foxf1^–^Kit^+^ ECs, *n* = 4; BLM-capEPCs, *n* = 4). (**E**) Lung resistance (R_rs_) and elastance (E_rs_) measured by flexiVent show improved respiratory mechanics in capEPCs-treated mice (control, *n* = 3; BLM-mock, *n* = 5; BLM-Foxf1^–^Kit^+^ ECs, *n* = 4; BLM-capEPCs, *n* = 4). (**F**) Transplantation of capEPCs increased inspiratory capacity (IC), static compliance (C_st_), and pressure-volume loop area, indicating enhanced lung compliance and elasticity (*n* = 3–5 mice per group). Data are presented as mean ± SD. Body weight recovery in **C** was analyzed by 2-way ANOVA with Greenhouse-Geisser correction (^#^*P* < 0.05, ^##^*P* < 0.01). Other comparisons were performed using 1-way ANOVA with Fisher’s LSD test. *P < 0.05, **P < 0.01, ***P < 0.001, ****P < 0.0001.

**Figure 4:**
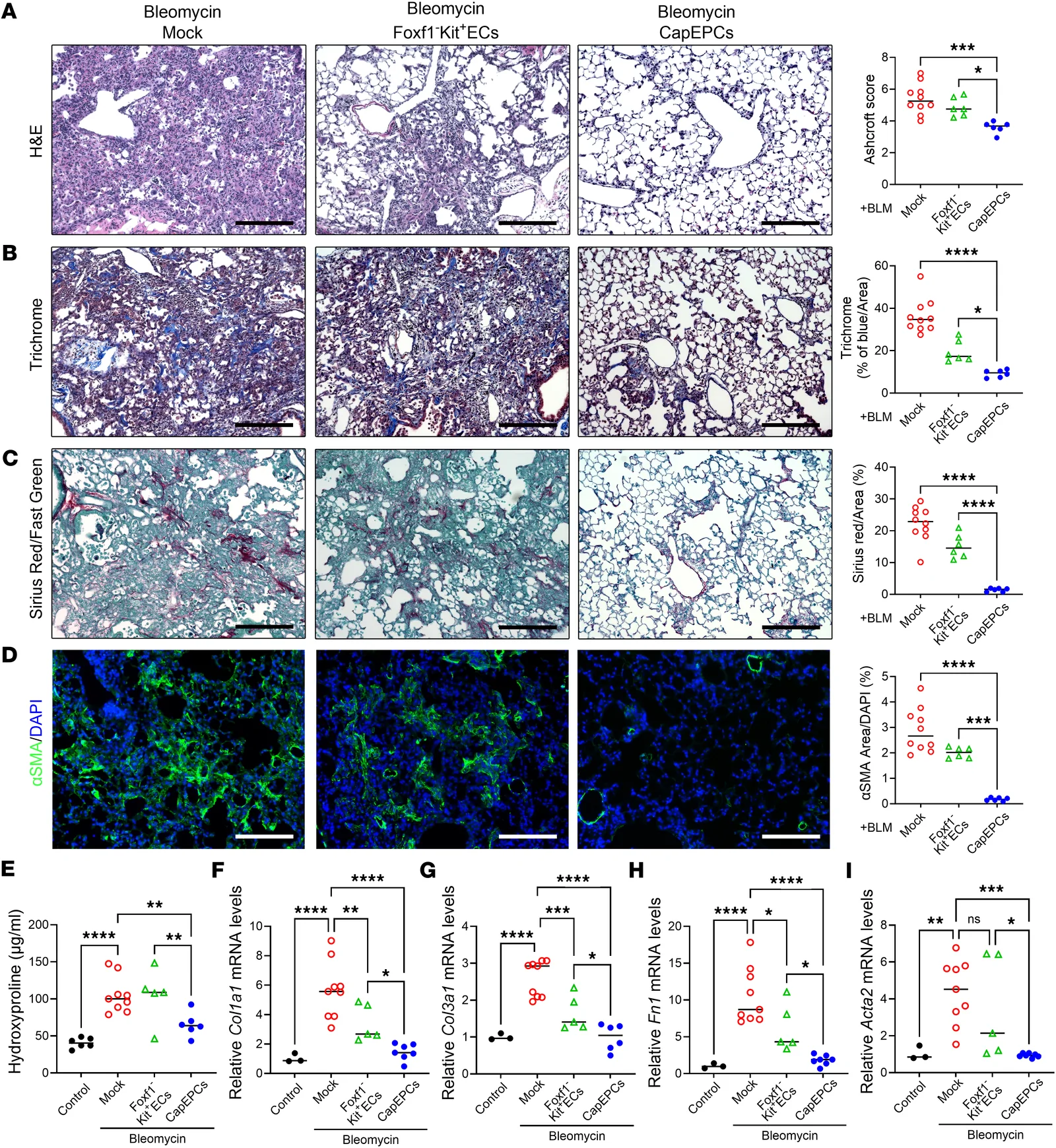
Transplantation of capEPCs attenuates fibrotic remodeling in bleomycin-treated mice. (**A**–**D**) Histological analysis of lungs collected 21 days after bleomycin injury shows reduced fibrotic remodeling following capEPC transplantation. Lung sections were stained with H&E (**A**), Masson’s trichrome (**B**), Sirius red/fast green (**C**), and immunofluorescence for αSMA (green) (**D**). Nuclei were counterstained with DAPI (blue). Right panels show quantitative analysis of Ashcroft score, trichrome-positive area, Sirius red–positive area, and αSMA-positive area. (BLM-mock, *n* = 10; BLM-Foxf1^–^Kit^+^ ECs, *n* = 6; BLM-capEPCs, *n* = 6.) Scale bars: 50 μm. (**E**) Hydroxyproline assay demonstrates reduced collagen deposition in lungs of capEPC-transplanted mice. *n* = 5–9 mice per group. (**F**–**I**) qRT-PCR analysis of total lung RNA reveals downregulation of fibrosis-associated genes in capEPC-transplanted mice. *Actb* was used for normalization. (Control group, *n* = 3; BLM-mock, *n* = 9; BLM-FOXF1^–^KIT^+^ ECs, *n* = 5; BLM-capEPCs. *n* = 7.) Data are presented as mean ± SD. One-way ANOVA with Fisher’s LSD test was used for all comparisons. *P < 0.05, **P < 0.01, ***P < 0.001, ****P < 0.0001.

**Figure 5:**
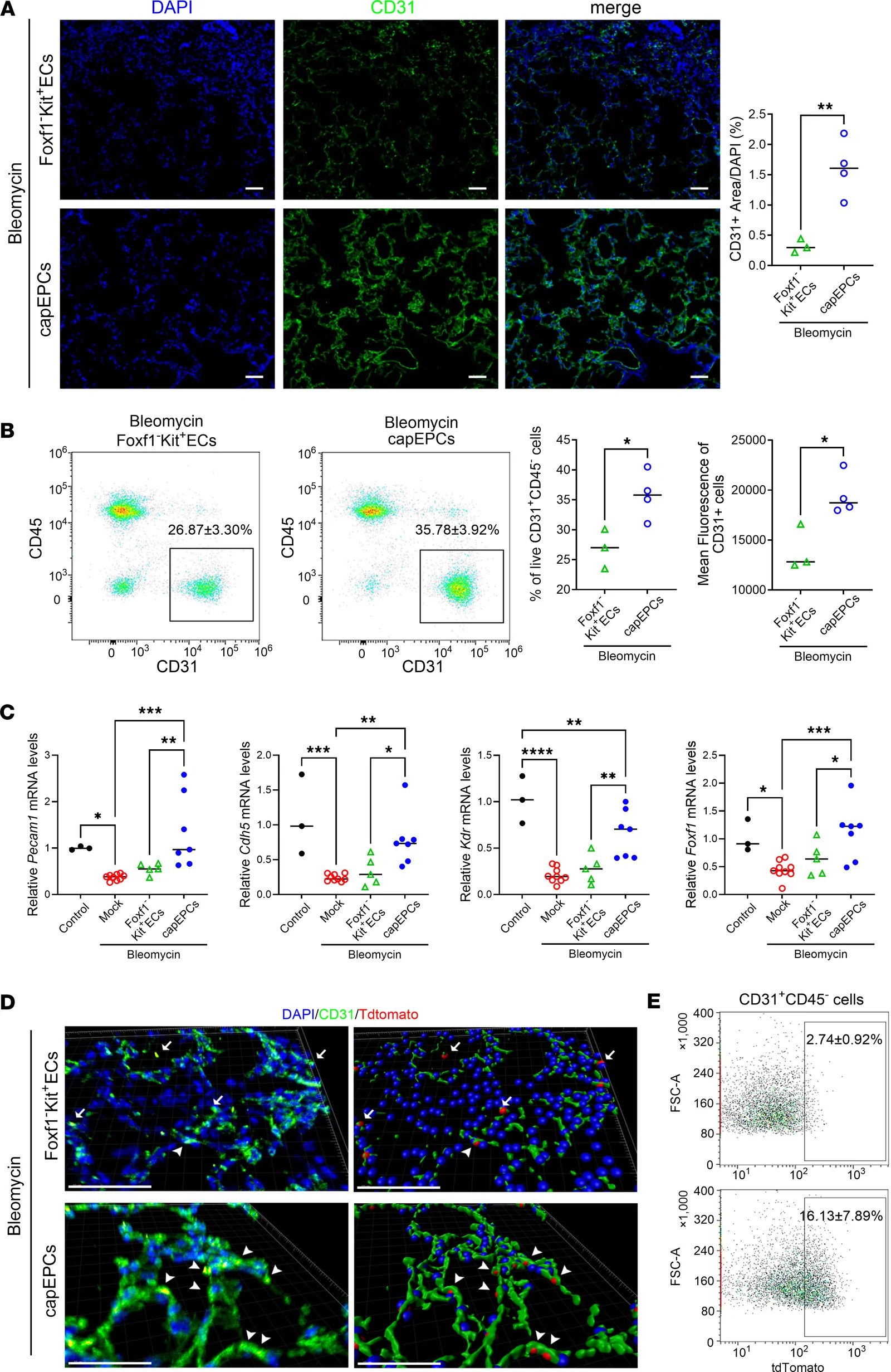
Transplantation of capEPCs increases the number of lung ECs in bleomycin-injured mice. (**A**) Left: Immunofluorescent staining of lung sections collected 21 days after bleomycin injury with CD31 (green) and DAPI (blue) shows increased endothelial coverage in capEPC-transplanted lungs compared with Foxf1^–^Kit^+^ EC–transplanted lungs. Right: Quantification of CD31^+^ area normalized to DAPI^+^ area (BLM-Foxf1^–^Kit^+^ ECs, *n* = 3; BLM-capEPCs, *n* = 4). Scale bars: 50 μm. (**B**) Flow cytometry analysis shows a higher percentage of ECs and increased CD31 mean fluorescence intensity of ECs in lungs of mice transplanted with capEPCs (BLM-Foxf1^–^Kit^+^ ECs, *n* = 3; BLM-capEPCs, *n* = 4). (**C**) qRT-PCR analysis of total lung RNA demonstrates increased *Pecam1*, *Cdh5*, *Kdr*, and *Foxf1* mRNAs in capEPC-treated lungs. *Actb* was used for normalization (control, *n* = 3; BLM-mock, *n* = 9; BLM-Foxf1^–^Kit^+^ ECs, *n* = 5; BLM-capEPCs, *n* = 7). (**D**) Confocal imaging of lungs shows increased integration of donor tdTomato^+^ cells within CD31^+^ vasculature in the capEPC-treated group. Arrowheads indicate CD31^+^tdTomato^+^ double-positive cells; arrows mark tdTomato^+^ single-positive donor cells. Scale bars: 10 μm. (**E**) Flow cytometry analysis at day 13 after transplantation shows a higher percentage of tdTomato^+^ cells within lung ECs of capEPC-transplanted lungs (BLM-Foxf1^–^Kit^+^ ECs, *n* = 3; BLM-capEPCs, *n* = 7). Data are presented as mean ± SD. Unpaired 1-tailed *t* tests were used in **A** and **B**; 1-way ANOVA with Fisher’s LSD test was used in **C**. *P < 0.05, **P < 0.01, ***P < 0.001, ****P < 0.0001.

**Figure 6:**
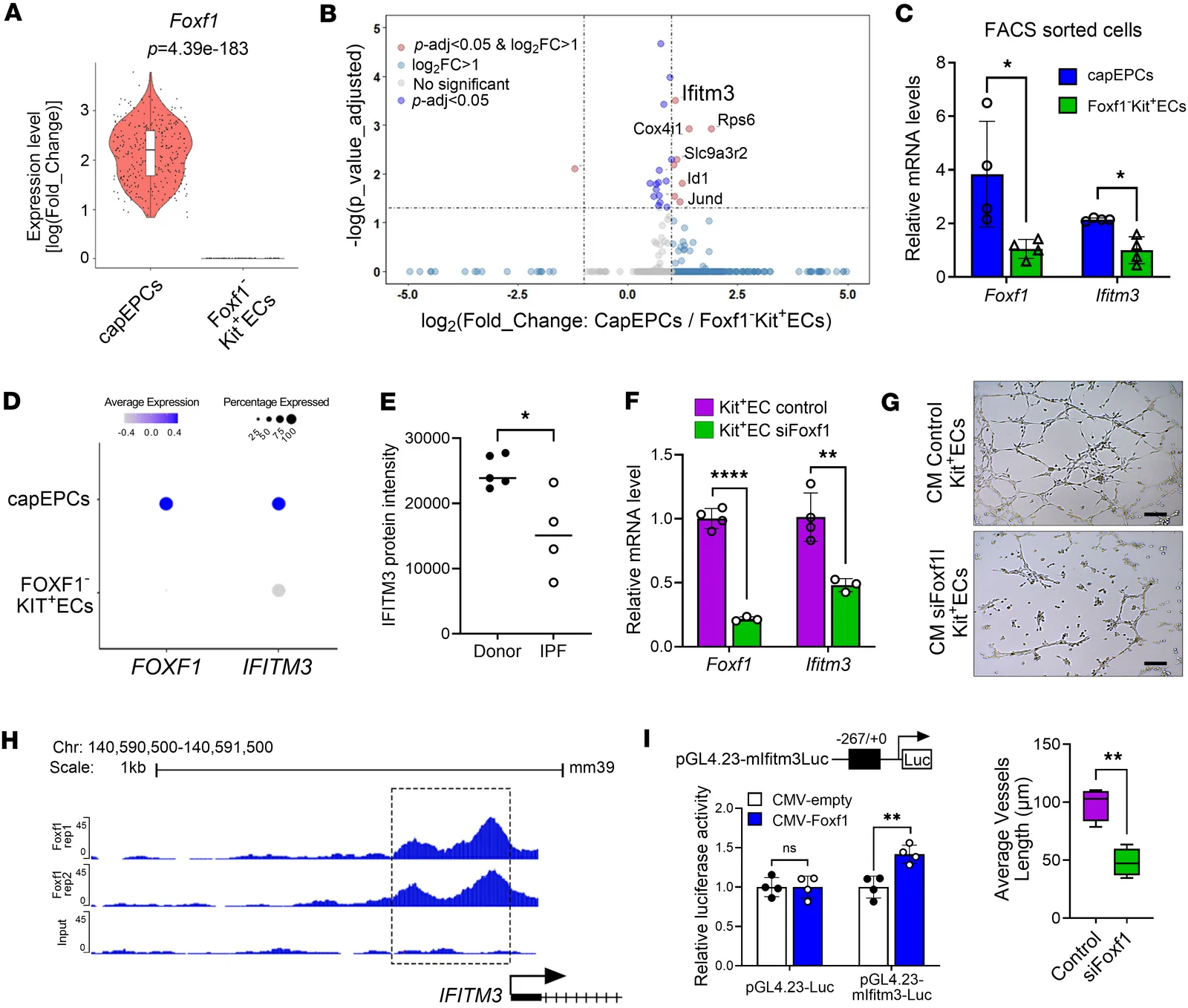
*Ifitm3* is a direct transcriptional target of FOXF1. (**A**) scRNA-seq of adult mouse lungs shows *Foxf1* mRNA in capEPCs (*n* = 312 cells) compared with Foxf1^–^Kit^+^ ECs (*n* = 70 cells). (**B**) Differentially expressed genes between capEPCs and Foxf1^–^Kit^+^ ECs show upregulation of *Ifitm3* in capEPCs. (**C**) qRT-PCR of FACS-sorted lung ECs shows higher *Foxf1* and *Ifitm3* mRNA levels in capEPCs (*n* = 4). (**D**) Dot plot analysis of human lung scRNA-seq data (GSE227136) demonstrates coexpression of *IFITM3* and *FOXF1* in capEPCs. (**E**) Quantitative proteomic analysis of bronchoalveolar lavage fluid (BALF) from donor controls (*n* = 5) and patients with idiopathic pulmonary fibrosis (IPF; *n* = 4) demonstrates decreased IFITM3 protein levels in IPF BALF samples. Data were extracted and reanalyzed from a previously published BALF proteomics dataset (31). A 2-tailed Mann-Whitney test was used. (**F** and **G**) siRNA-mediated knockdown of Foxf1 in Kit^+^ lung ECs decreased *Foxf1* and *Ifitm3* mRNA expression (**F**; control, *n* = 4; siFoxf1, *n* = 3), and conditioned medium from these cells reduced endothelial tube formation in vitro (**G**; *n* = 4 per group). Scale bars in **G**: 100 μm. (**H**) ChIP-seq analysis (GSE77951) shows FOXF1 binding to the *Ifitm3* promoter region. (**I**) Luciferase (Luc) assay demonstrates increased transcription from *Ifitm3* promoter region (–267/+0 bp) in the presence of FOXF1 (*n* = 4). Data are presented as mean ± SD. Unpaired 2-tailed *t* tests were used. *P < 0.05, **P < 0.01, ****P < 0.0001.

**Figure 7:**
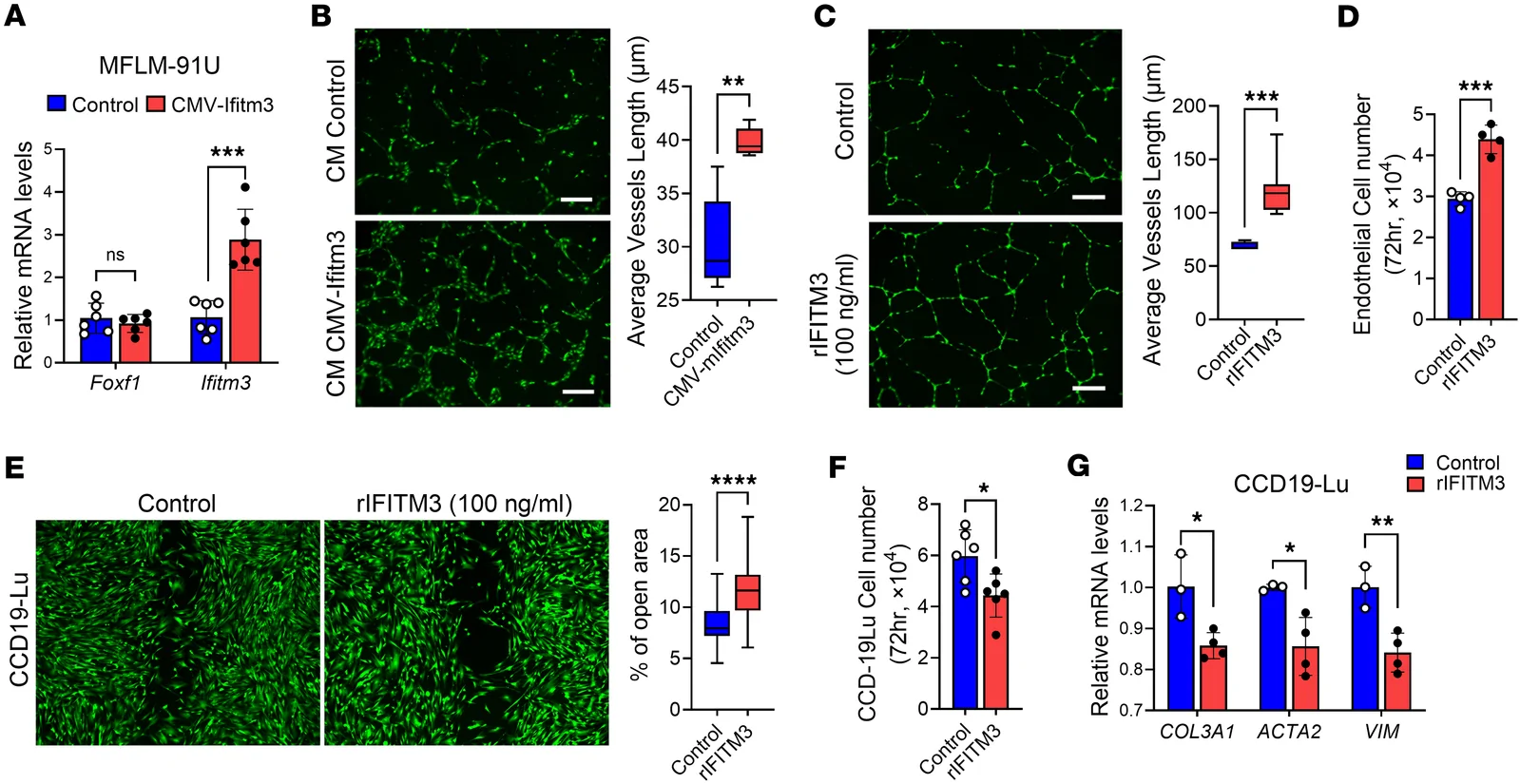
IFITM3 promotes EC functions but inhibits fibroblast activation. (**A**) qRT-PCR analysis confirms increased Ifitm3 expression in MFLM-91U cells transfected with CMV-Ifitm3 (*n* = 6). (**B**) Conditioned medium (CM) from IFITM3-overexpressing MFLM-91U cells enhances endothelial tube formation in vitro (*n* = 5). (**C**) Recombinant IFITM3 protein (rIFITM3; 100 ng/mL) promotes tube formation in cultured HPAECs (*n* = 7). (**D**) rIFITM3 increases HPAEC proliferation at 72 hours (*n* = 4). (**E**) Cell migration assay using a 2-well culture insert demonstrates reduced migration of CCD-19Lu fibroblasts following rIFITM3 treatment (*n* = 6 independent wells per group). Scale bars in **B** and **C**: 100 μm. Images in **E** were acquired using a 10× objective. (**F**) rIFITM3 suppresses fibroblast proliferation after 72 hours (*n* = 6). (**G**) qRT-PCR shows reduced expression of profibrotic *COL3A1*, *ACTA2*, and *VIM* after rIFITM3 treatment (control, *n* = 3; rIFITM3, *n* = 4 for each gene). Data are presented as mean ± SD. Unpaired 2-tailed *t* tests were used. *P < 0.05, **P < 0.01, ***P < 0.001, ****P < 0.0001.

**Figure 8:**
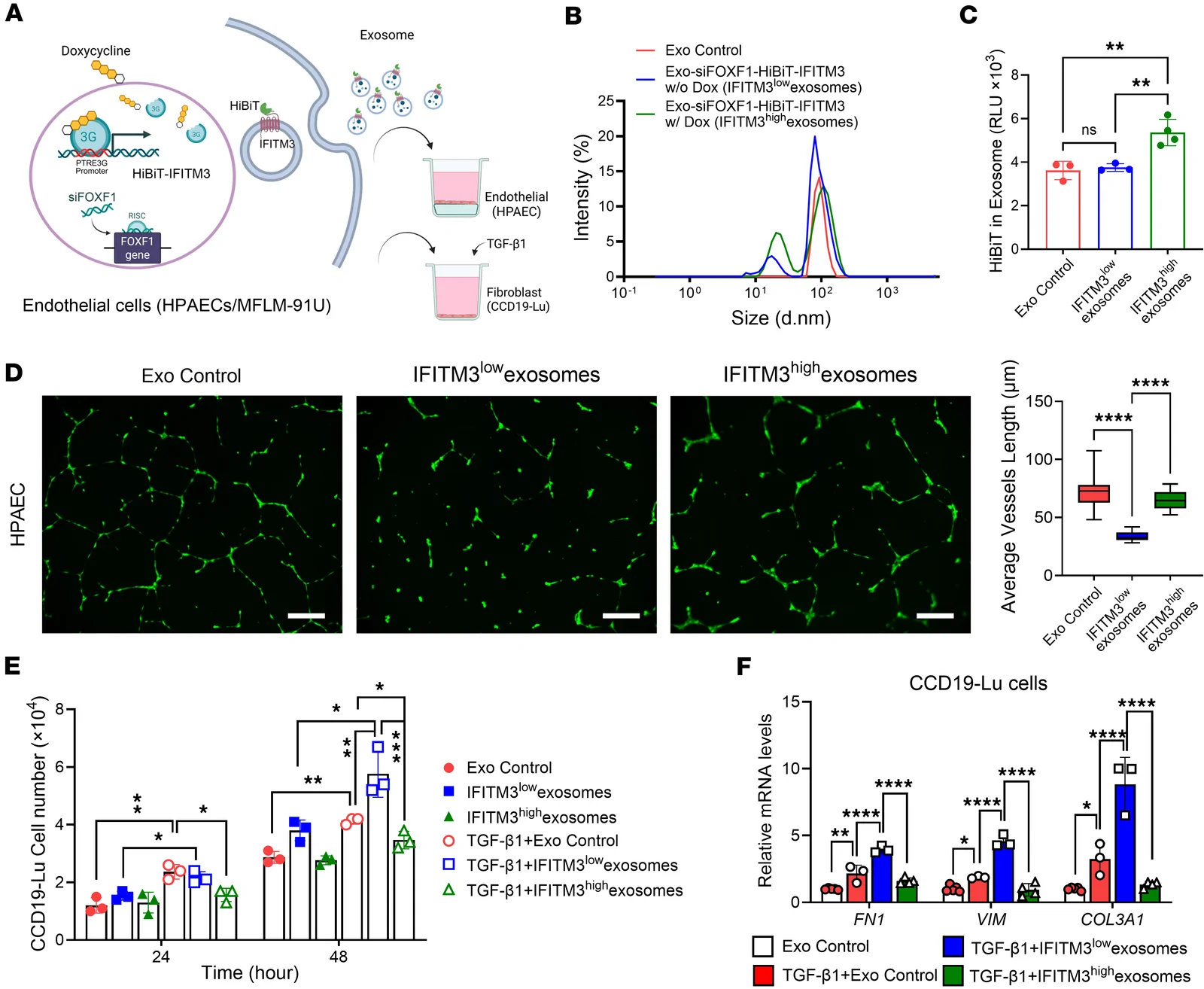
Treatment with IFITM3-containing exosomes improves EC functions but inhibits fibroblast activation. (**A**) Schematic diagram illustrating generation of ECs expressing exogenous IFITM3 fused to a HiBiT tag under doxycycline-inducible control. FOXF1 siRNA was used to suppress endogenous IFITM3 expression. Doxycycline (Dox) treatment induced HiBiT-IFITM3 expression, and conditioned media were collected for exosome isolation. Isolated exosomes were subsequently applied to endothelial cells (HPAECs) or fibroblasts (CCD-19Lu). (**B**) Size distribution of isolated exosomes measured by dynamic light scattering, showing similar particle profiles across groups (Exo Control, *n* = 3; IFITM3^lo^ exosomes, *n* = 8; IFITM3^hi^ exosomes, *n* = 5 independent exosome preparations). (**C**) Nano-Glo HiBiT extracellular detection assay shows increased HiBiT-IFITM3 signal in exosomes from Dox-induced cells (IFITM3^hi^ exosomes; *n* = 4) compared with non-induced controls (IFITM3^lo^ exosomes; *n* = 3). (**D**) Left: Endothelial tube formation assay in HPAECs shows impaired angiogenesis following treatment with IFITM3^lo^ exosomes, while IFITM3^hi^ exosomes restore tube-forming ability. Scale bars: 100 μm. Right: Quantification of vessel length (*n* = 12). Boxes indicate the 25th to 75th percentiles, center lines indicate the medians, and whiskers indicate the minimum and maximum values. (**E**) Cell proliferation assay using CCD-19Lu fibroblasts shows that IFITM3^hi^ exosomes suppress TGF-β1–induced fibroblast proliferation at 24 and 48 hours, compared with IFITM3^lo^ exosomes (*n* = 3). (**F**) qRT-PCR analysis demonstrates that treatment with IFITM3^hi^ exosomes suppresses TGF-β1–induced expression of the profibrotic genes *FN1*, *VIM*, and *COL3A1* in CCD-19Lu fibroblasts (control, *n* = 5; Exo Control + TGF-β1, *n* = 3; IFITM3^lo^ exosomes + TGF-β1, *n* = 3; IFITM3^hi^ exosomes + TGF-β1, *n* = 4 for each gene). Data are presented as mean ± SD. One-way ANOVA with Fisher’s LSD test was used for multiple comparisons. *P < 0.05, **P < 0.01, ***P < 0.001, ****P < 0.0001.

**Figure 9:**
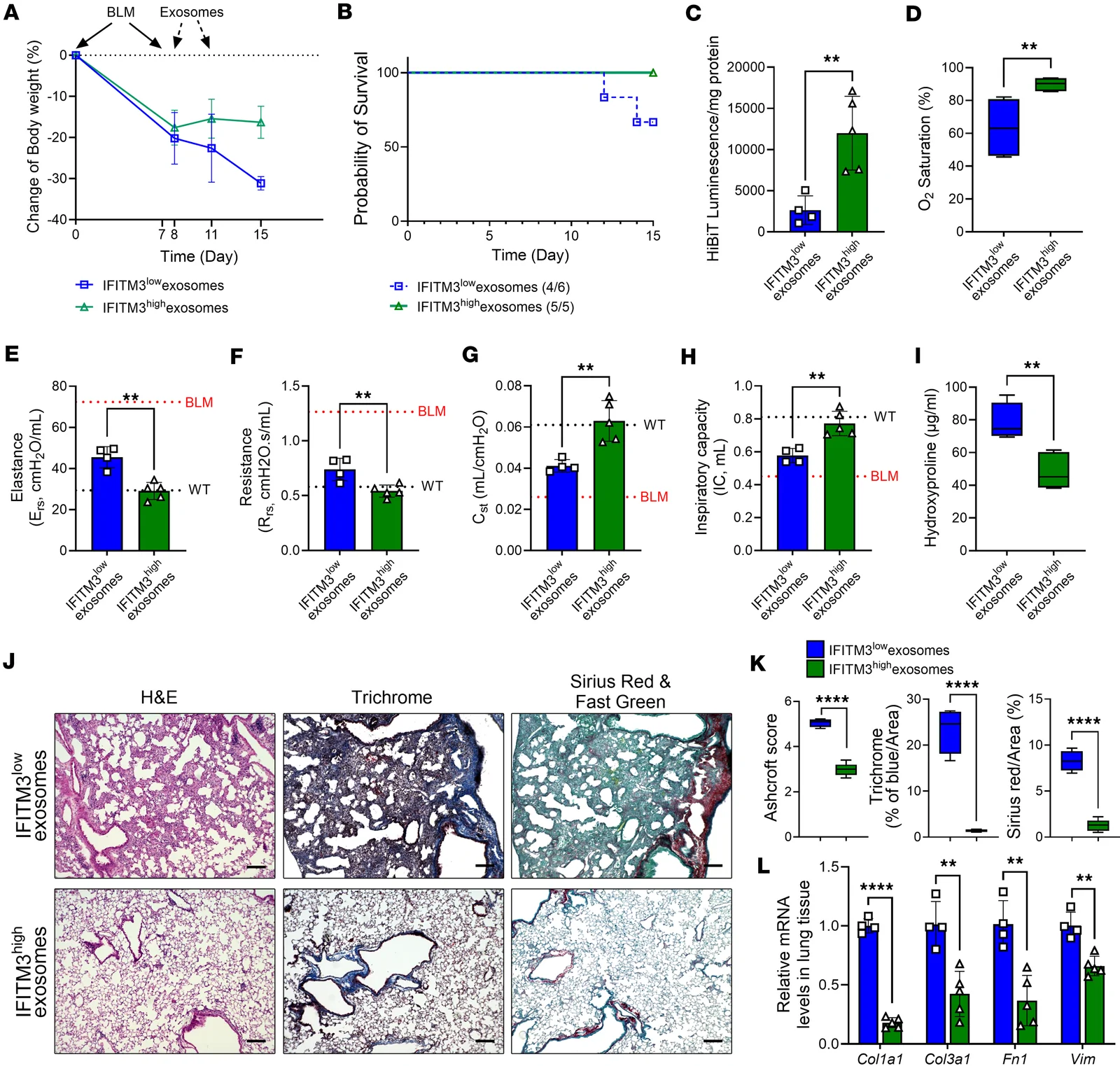
Treatment with IFITM3-containing exosomes decreases PF and improves survival in bleomycin-injured mice. (**A**) Treatment with IFITM3-containing (IFITM3^hi^) exosomes attenuated body weight loss in mice after bleomycin (BLM) injury. Exosomes were delivered on days 8 and 11 after BLM treatment. (**B**) Survival was improved in mice treated with IFITM3^hi^ exosomes compared with mice treated with IFITM3^lo^ exosomes. Numbers in parentheses indicate surviving mice/total mice per group. (**C**) HiBiT luminescence in lung lysates on day 15 post-bleomycin confirmed presence of IFITM3-containing exosomes in lung tissue (IFITM3^lo^, *n* = 4; IFITM3^hi^, *n* = 5). (**D**) Oxygen saturation was increased in mice treated with IFITM3^hi^ exosomes (IFITM3^lo^, *n* = 4; IFITM3^hi^, *n* = 6). (**E**–**H**) FlexiVent measurements on day 15 showed that IFITM3^hi^ exosomes improved elastance (E_rs_) (**E**), resistance (R_rs_) (**F**), static compliance (C_st_) (**G**), and inspiratory capacity (IC) (**H**). Red and black dotted lines represent the average values of the bleomycin and wild-type (WT) groups, respectively. (**I**) Hydroxyproline assay revealed reduced collagen deposition in mice treated with IFITM3^hi^ exosomes (IFITM3^lo^, *n* = 4; IFITM3^hi^, *n* = 5). (**J**) Representative H&E, Masson’s trichrome, and Sirius red/fast green staining of lungs collected on day 15. Scale bars: 100 μm. (**K**) Quantification of Ashcroft scores and trichrome and Sirius red areas from **J**. (**L**) qRT-PCR showed reduced *Col1a1*, *Col3a1*, *Fn1*, and *Vim* in the IFITM3^hi^ exosome–treated mice. Expression was normalized to *Actb*. Data are presented as mean ± SD. IFITM3^hi^ exosome–treated mice, *n* = 5; IFITM3^lo^ exosome–treated mice, *n* = 4. For box-and-whisker plots in **D**, **I**, and **K**, boxes indicate the 25th to 75th percentiles, center lines indicate the medians, and whiskers indicate the minimum and maximum values. Unpaired 2-tailed *t* tests were used in **C**–**I**, **K**, and **L**. **P < 0.01, ****P < 0.0001.

## Version 1 10/08/2026

Electronic publication
