# Supplementary material for: Transplantation of endothelial progenitor cells attenuates pulmonary fibrosis via IFITM3-containing exosomes
Source: JCI Insight. 2026 Oct 8;11(19):e199743. doi: 10.1172/jci.insight.199743 (PMC13726431; doi:10.1172/jci.insight.199743)
Supplement: Supplementary file 1 [file jciinsight-11-199743-s293.pdf]

Supplemental Figure S1. Single-cell RNA-seq analysis reveals endothelial cell heterogeneity and endothelial subtype redistribution in human pulmonary fibrosis lungs.

(**A**) UMAP plots showing five endothelial subclusters identified from donor, “less fibrotic,” and “more fibrotic” human lungs using scRNA-seq dataset GSE227136. Endothelial populations include general capillary endothelial cells (CAP1), aerocyte capillary endothelial cells (CAP2), arterial endothelial cells (AEC), venous endothelial cells (VEC), and systemic venous endothelial cells (SVEC).

(**B**) Dot plot showing representative marker gene expression across endothelial subclusters. Dot size indicates the percentage of expressing cells, and color scale represents average expression level.

(**C**) Donor-level analysis of endothelial subtype distribution across donor, less fibrotic, and more fibrotic lungs. Percentages of each endothelial subtype were calculated relative to total endothelial cells in each sample. Each point represents an individual donor sample. Statistical significance was assessed using Kruskal–Wallis test followed by Benjamini–Hochberg-adjusted pairwise Wilcoxon comparisons. ***$P < 0.001$, ****$P < 0.0001$.

(**D**) Enrichr pathway analysis of genes upregulated in capEPCs compared with other capillary ECs identifies stemness- and KIT-associated signaling pathways. Red boxes highlight selected pathways.

(**E**) Heatmap showing differentially expressed genes (adjusted $P < 0.05$) in capEPCs between donor and fibrotic lungs. Color scale represents Z-scored expression values.

(**F**) UMAP feature plots showing *FOXF1* and *KIT* expression in donor, less fibrotic, and more fibrotic lungs. Red color intensity indicates relative transcript expression levels. Data were obtained from previously published scRNA-seq dataset GSE227136 and reanalyzed in the current study.

Supplemental Figure S2. Single-cell RNA-seq analysis reveals endothelial heterogeneity in fibrotic mouse lungs.

(**A**) UMAP plots of endothelial subclusters in untreated and bleomycin-treated mouse lungs using published datasets GSE264162 and GSE264151. Subclusters included general capillary endothelial cells (CAP1), aerocyte capillary endothelial cells (CAP2), arterial endothelial cells (AEC), and venous endothelial cells (VEC).

(**B**) Dot plot of representative marker genes across endothelial subclusters. Dot size indicates the percentage of expressing cells, and color intensity indicates

average expression.

(**C**) Enrichr analysis of genes upregulated in capEPCs compared with other capillary ECs. Red boxes highlight selected KIT- and stemness-associated pathways.

(**D**) Heatmap of differentially expressed genes (adjusted P < 0.05) in capEPCs from control and bleomycin-treated lungs. Colors indicate Z-scored expression.

(**E**) UMAP feature plots of Foxf1 and Kit expression in control and bleomycin-treated lungs using GSE264162 and GSE264151.

(**F**) Percentage of capEPCs (Foxf1$^+$Kit$^+$ endothelial cells) among total endothelial cells across days 0, 7, 10, 21, and 28 after bleomycin injury using GSE141259.

(**G**) Endothelial vascular and mesenchymal fibrosis gene module scores across bleomycin injury time points. Each dot represents one mouse lung sample (n = 2–7 per group); bars indicate mean ± SD. Statistical significance was assessed by 1-way ANOVA with post hoc multiple-comparison tests. P < 0.05, P < 0.01, P < 0.001, P < 0.0001; ns, not significant.

(**H**) Representative flow cytometry plots and quantification of CD117$^+$CD45$^+$ cells among total live lung cells on day 21 after bleomycin injury. Control n = 3; bleomycin n = 7. Bars indicate mean ± SD, and points represent biological replicates. An unpaired 2-tailed t test was used; ns, not significant.

Supplemental Figure S3. Reduced cKIT$^+$ endothelial cells in bleomycin-injured fibrotic lungs.

Representative immunofluorescence images showing CD31 (green) and Kit (red) staining in control and day 21 post-bleomycin fibrotic mouse lungs, demonstrating reduced numbers of Kit$^+$ endothelial cells in fibrotic lungs. Images are reproduced from Figure 2E. Scale bar, 100 μm.

Supplemental Figure S4. CapEPC transplantation restores epithelial gene expression and reduces pathological fibroblast accumulation following bleomycin-induced lung injury.

(**A**) Relative mRNA expression levels of epithelial markers *Epcam* and *Cdh1* in whole-lung RNA isolated from control mice and bleomycin-injured mice receiving mock treatment, Foxf1$^-$Kit$^+$ endothelial cells (ECs), or capEPCs transplantation. Bleomycin was administered on days 0 and 7, followed by EPC transplantation on day 8. Lungs were collected on day 21 for RNA analysis. CapEPCs transplantation significantly increased *Epcam* and *Cdh1* expression compared with mock-treated bleomycin lungs, whereas Foxf1$^-$Kit$^+$ EC

transplantation showed limited recovery.

(**B**) Representative immunofluorescence images of lung sections from bleomycin-injured mice treated with Foxf1$^-$Kit$^+$ ECs or capEPCs. Sections were stained for CTHRC1 (green) and counterstained with DAPI (blue). capEPCs-treated lungs showed reduced accumulation of CTHRC1$^+$ pathological fibroblasts compared with Foxf1$^-$Kit$^+$ EC-treated lungs. Scale bar, 100 μm.

(**C**) Flow cytometric analysis of immune cell infiltration in lung single-cell suspensions from control lungs, bleomycin-injured lungs, and bleomycin-injured lungs treated with Foxf1$^-$Kit$^+$ ECs or capEPCs. Representative flow cytometry plots show CD45 and CD31 staining profiles. Immune cells were defined as CD45$^+$CD31$^-$ leukocytes, and the lower panel shows quantification of CD45$^+$CD31$^-$ immune cells among total live lung cells.

Each point represents an individual mouse (n = 3–9 per group). Bars indicate mean ± SD. Statistical significance was assessed using one-way ANOVA with post hoc multiple-comparison tests. *P < 0.05, **P < 0.01, ***P < 0.001, ****P < 0.0001; ns, not significant.

Supplemental Figure S5. CapEPC transplantation increases endothelial proliferation and reduces endothelial apoptosis in bleomycin-injured lungs.

(**A**) Representative immunofluorescence images of lung sections harvested on day 21 after bleomycin injury from mice receiving Foxf1$^-$Kit$^+$ endothelial cells (ECs) or capEPC transplantation. Sections were stained for Ki67 (red), Endomucin (green), and DAPI (blue) to identify proliferating endothelial cells. capEPC-treated lungs showed increased numbers of Ki67$^+$ endothelial cells within Endomucin-positive vascular structures (arrows) compared with Foxf1$^-$Kit$^+$ EC-treated lungs.

(**B**) Representative immunofluorescence images of lung sections harvested on day 21 after bleomycin injury and stained for Cleaved Caspase-3 (green), Endomucin (red), and DAPI (blue) to identify apoptotic endothelial cells. Bleomycin-injured lungs receiving Foxf1$^-$Kit$^+$ ECs showed increased Cleaved Caspase-3 staining within vascular structures, whereas capEPC-treated lungs exhibited reduced endothelial apoptosis.

Merged images illustrate proliferating or apoptotic endothelial cells within the pulmonary vasculature. Images are representative of multiple lung samples analyzed per group. Scale bars, 100 μm.

Supplemental Figure S6. IFITM3 expression is reduced in fibrotic human and mouse lungs and promotes endothelial angiogenic activity.

(**A**) Representative immunofluorescence images of donor and pulmonary fibrosis (PF) human lung sections stained for DAPI (gray), KIT (red), IFITM3 (green), and FOXF1 (blue). In donor lungs, IFITM3 signal colocalized with $KIT^{+}FOXF1^{+}$ endothelial progenitor cells within alveolar capillary regions. In PF lungs, IFITM3 expression and $KIT^{+}FOXF1^{+}$ cells were reduced.
(**B**) Representative immunofluorescence images of control and day 21 post-bleomycin mouse lungs stained for DAPI (gray), KIT (red), IFITM3 (green), and FOXF1 (blue). Control lungs showed IFITM3 expression within alveolar capillary structures and partial colocalization with $KIT^{+}Foxf1^{+}$ endothelial progenitor cells, whereas bleomycin-treated lungs exhibited reduced IFITM3 signal and fewer $KIT^{+}Foxf1^{+}$ endothelial cells. Merged images are shown in the rightmost panels. Scale bars, 100 μm.
(**C**) Representative images of endothelial tube formation assays performed for 6 hours using human pulmonary microvascular endothelial cells (HPMECs) treated with recombinant human IFITM3 (rhIFITM3). rhIFITM3 treatment enhanced endothelial tube formation compared with untreated controls. Scale bar, 100 μm.
(**D**) Quantification of average tube length from endothelial tube formation assays shown in panel C (n = 5).
(**E**) HPMEC proliferation assay showing increased cell numbers following rhIFITM3 treatment at 24 and 48 hours compared with untreated controls (n = 4).
(**F**-**G**) SignalP-based prediction of signal peptide and transmembrane domains in human and mouse IFITM3 proteins. Upper panels show predicted protein topology indicating non-cytoplasmic, cytoplasmic, and transmembrane domains. Lower panels show SignalP 6.0 output plots demonstrating low probability of signal peptide sequences in human IFITM3 (UniProt Q01628) and mouse IFITM3 (UniProt Q9CQW9).
Data are presented as mean ± SD. Statistical significance was assessed using unpaired two-tailed t tests. **P < 0.01.

Supplemental Figure S7. Localization of IFITM3-containing exosomes and restoration of Ifitm3 expression in bleomycin-injured lungs.
(**A**) Representative immunofluorescence images of lung sections collected on day 15 after bleomycin injury and stained for endogenous IFITM3 (red), HiBiT (green), and DAPI (blue). Mice received $IFITM3^{low}$ or $IFITM3^{high}$ exosomes (20 μg in 50 μL PBS) on days 8 and 11 after bleomycin administration. Lungs treated with $IFITM3^{low}$ exosomes showed minimal HiBiT signal, whereas

IFITM3^high exosome-treated lungs demonstrated increased HiBiT localization within injured lung regions. Merged images show spatial overlap between endogenous IFITM3 and HiBiT-labeled IFITM3. Scale bars, 100 μm.

(**B**) Representative immunofluorescence images of lung sections collected on day 15 after bleomycin injury from mice treated with IFITM3^{low} or IFITM3^{high} exosomes (20 μg in 50 μL PBS administered on days 8 and 11 after bleomycin). Upper panels were stained for PDGFRα (cyan), CD31 (green), HiBiT (red), and DAPI (blue). Lower panels were stained for pro-SPC (cyan), T1α (green), HiBiT (red), and DAPI (blue). HiBiT-positive signals indicate localization of exosome-delivered IFITM3 within injured lung tissues. Scale bars, 50 μm.

Supplemental Figure S8. Distinct spatial distribution of KIT$^+$FOXF1$^+$ capEPCs and ABCG2$^+$ endothelial cells in human and mouse lungs.

(**A**) UMAP visualization of endothelial cells from the human lung scRNA-seq dataset GSE227136 showing the distribution of KIT$^+$FOXF1$^+$ endothelial progenitor cells (capEPCs) and ABCG2$^+$ endothelial cells across control, less fibrotic, and more fibrotic lungs. Grey dots represent all endothelial cells, and red dots indicate cells expressing the indicated markers. KIT$^+$FOXF1$^+$ capEPCs were primarily localized within general capillary endothelial cell clusters, whereas ABCG2$^+$ endothelial cells were enriched in venous and systemic venous endothelial populations.

(**B**) UMAP visualization of endothelial cells from mouse lung scRNA-seq datasets GSE264162 and GSE264151 showing the distribution of Foxf1$^+$Kit$^+$ capEPCs and Abcg2$^+$ endothelial cells in control and bleomycin-treated lungs. Grey dots represent all endothelial cells, and red dots indicate cells expressing the indicated markers. Foxf1$^+$Kit$^+$ capEPCs were predominantly localized within capillary endothelial populations, whereas Abcg2$^+$ endothelial cells were enriched in venous and non-capillary endothelial clusters.

These analyses demonstrate minimal spatial overlap between KIT$^+$FOXF1$^+$/Foxf1$^+$Kit$^+$ capEPCs and ABCG2$^+$/Abcg2$^+$ endothelial cells, supporting that these represent distinct endothelial progenitor populations in human and mouse lungs.

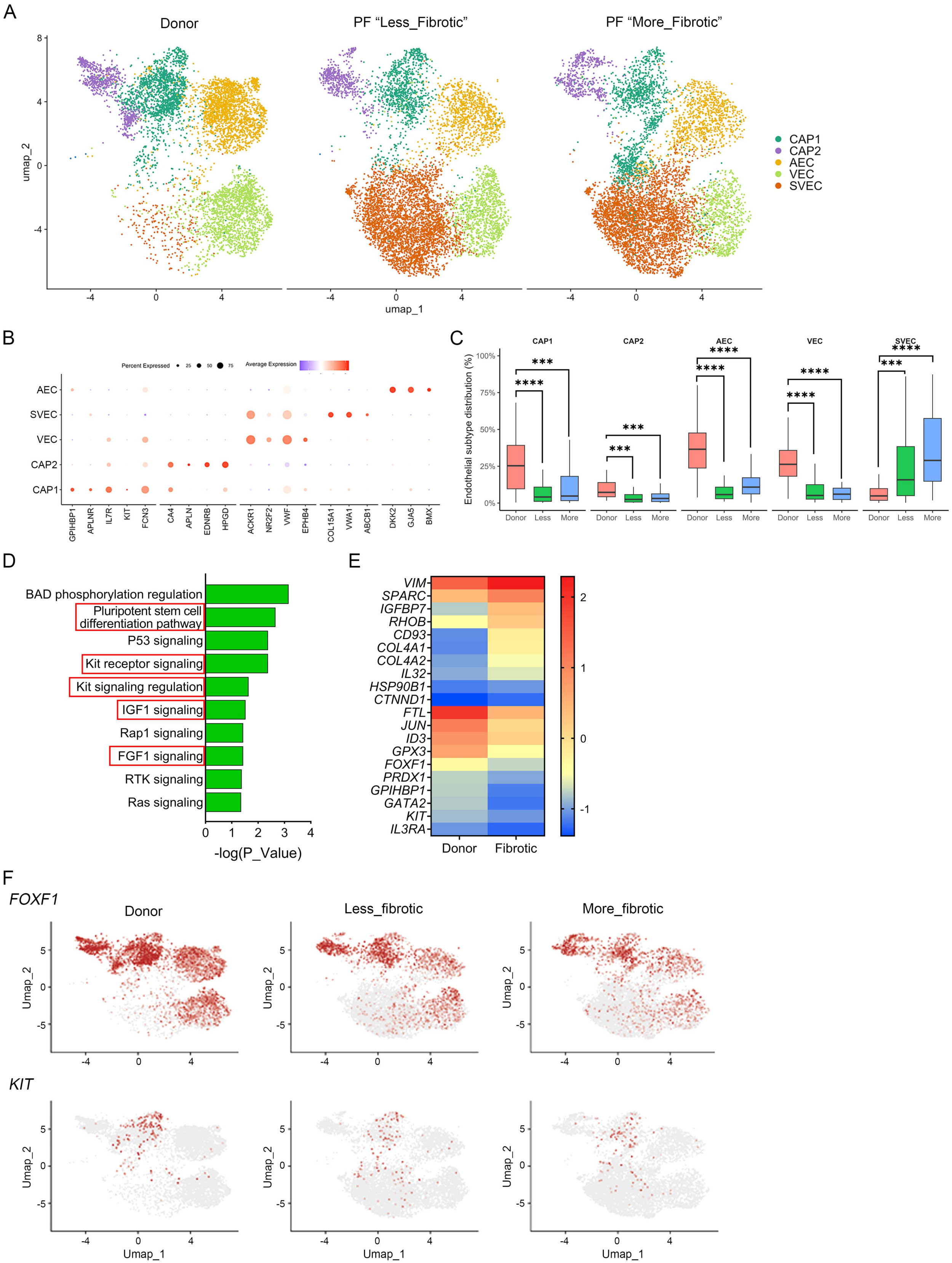
A
Donor
PF "Less_Fibrotic"
PF "More_Fibrotic"
umap_2
umap_1
CAP1
CAP2
AEC
VEC
SVEC
B
Percent Expressed
Average Expression
AEC
SVEC
VEC
CAP2
CAP1
GPIHBP1
APLNR
IL7R
KIT
FCN3
CA4
APLN
EDNRB
HPGD
ACKR1
NR2F2
VWF
EPHB4
COL15A1
VWA1
ABCB1
DKK2
GJA5
BMX
C
Endothelial subtype distribution (%)
Donor
Less
More
D
BAD phosphorylation regulation
Pluripotent stem cell differentiation pathway
P53 signaling
Kit receptor signaling
Kit signaling regulation
IGF1 signaling
Rap1 signaling
FGF1 signaling
RTK signaling
Ras signaling
-log(P_Value)
E
VIM
SPARC
IGFBP7
RHOB
CD93
COL4A1
COL4A2
IL32
HSP90B1
CTNND1
FTL
JUN
ID3
GPX3
FOXF1
PRDX1
GPIHBP1
GATA2
KIT
IL3RA
Donor
Fibrotic
F
FOXF1
Donor
Less_fibrotic
More_fibrotic
KIT
Umap_2
Umap_1

Lan et al. Sup Fig S2

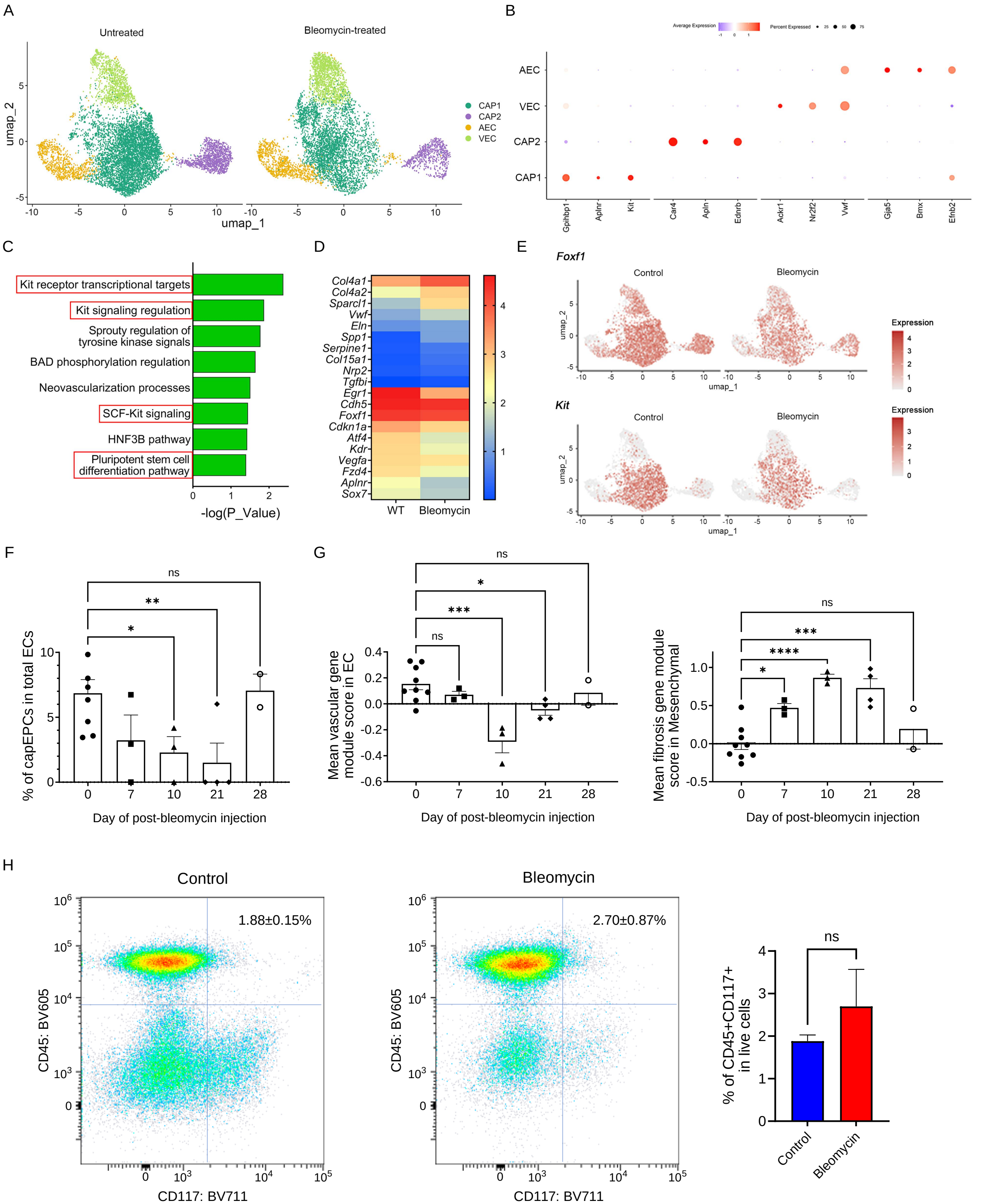

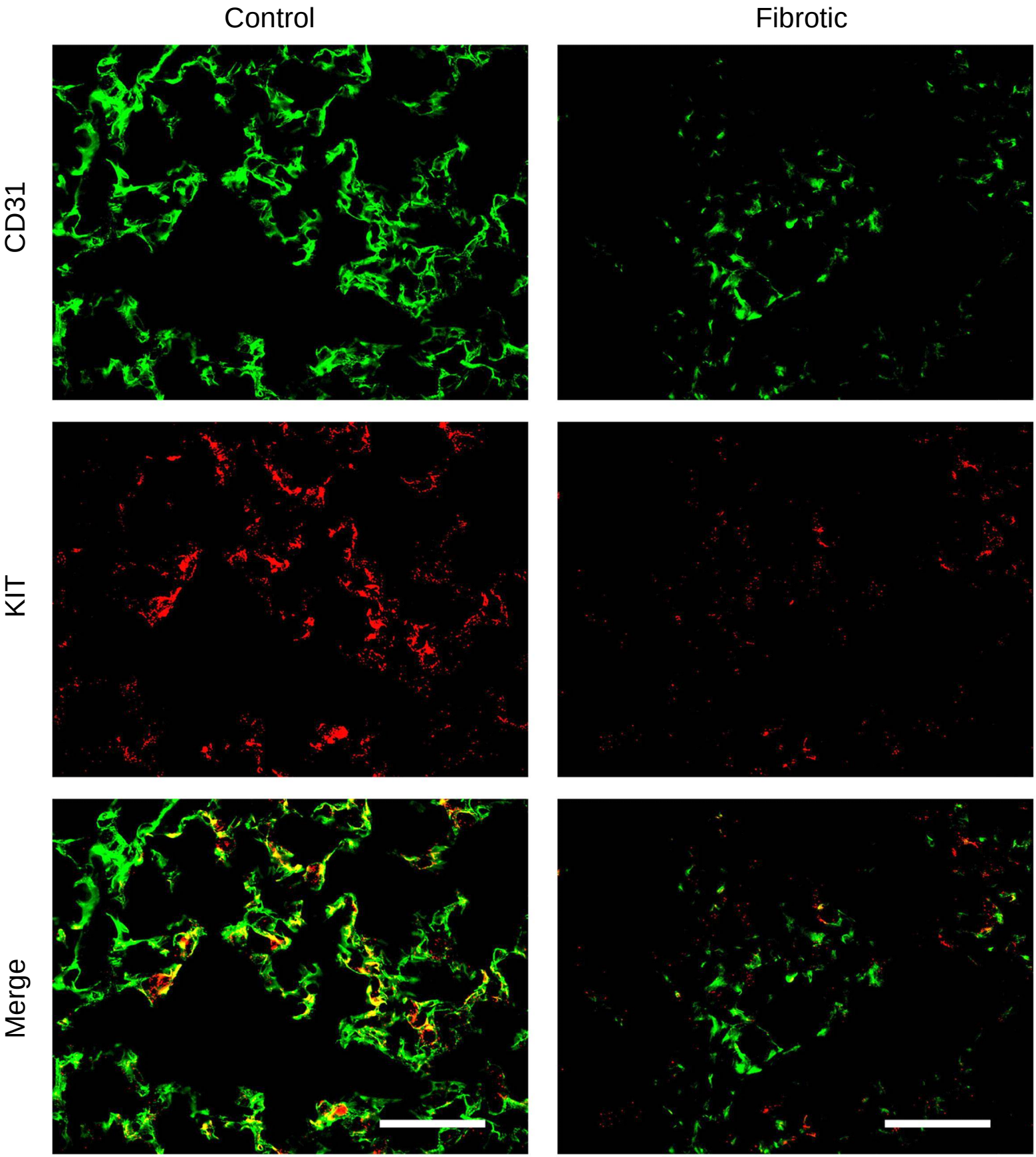
Control
Fibrotic
CD31
KIT
Merge

Lan et al. Sup Fig S4

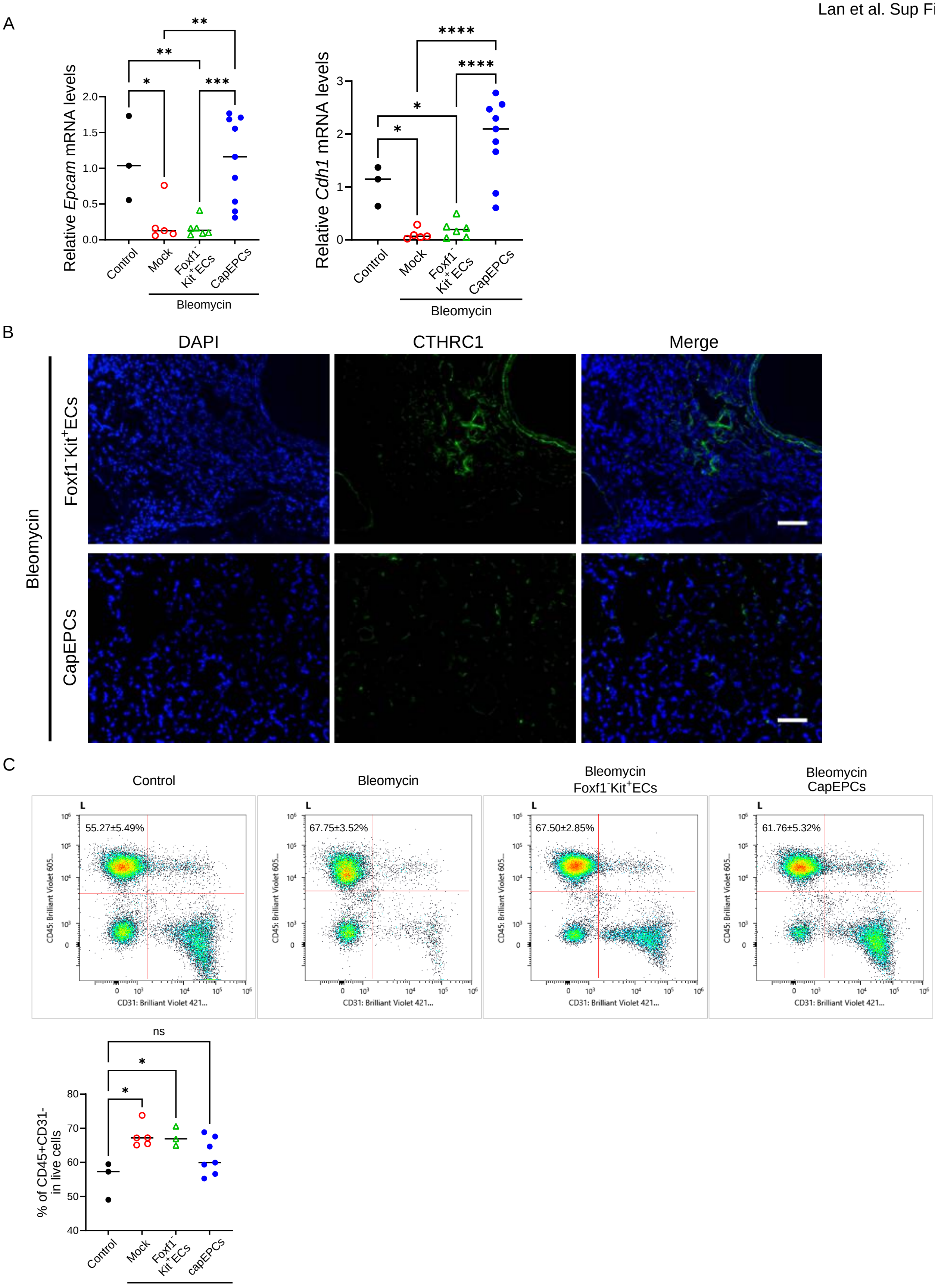

A

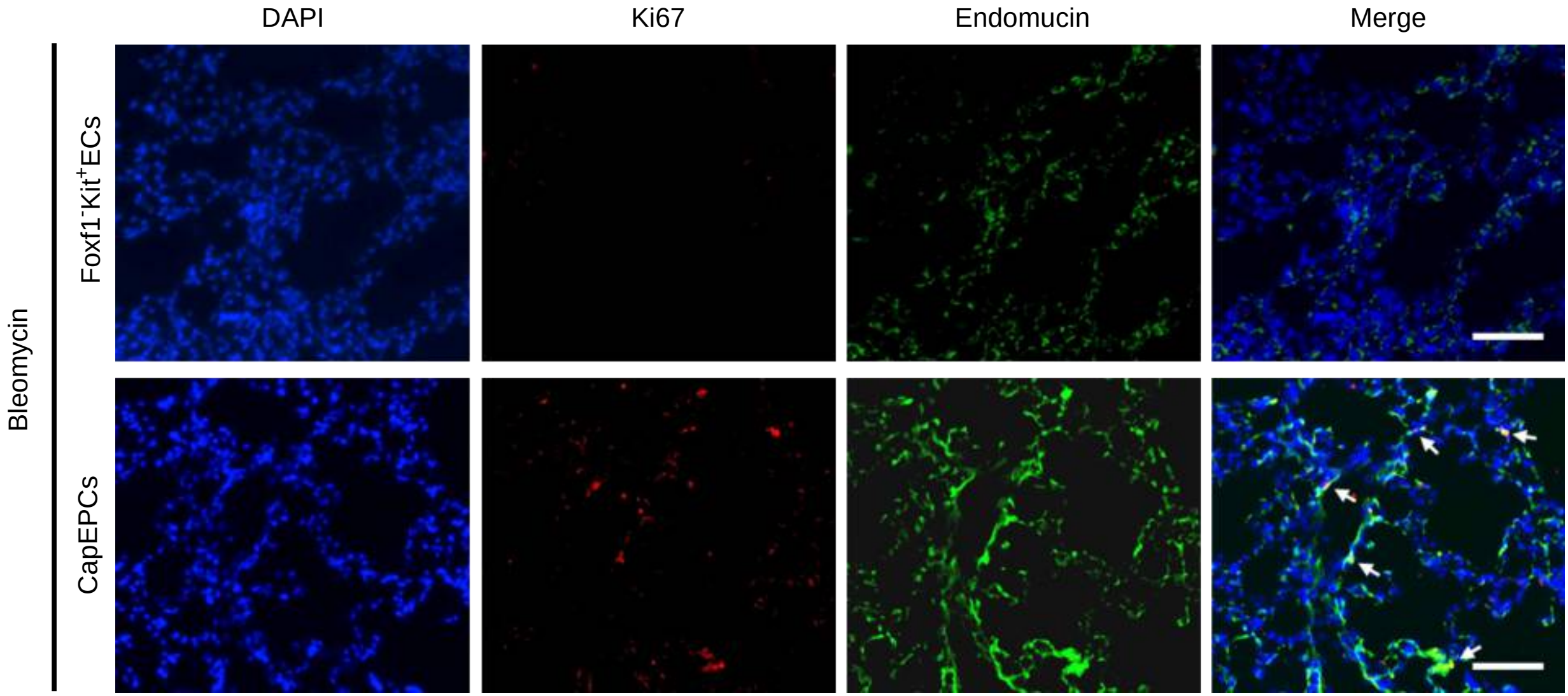
DAPI
Ki67
Endomucin
Merge
Bleomycin
Foxf1⁻Kit⁺ECs
CapEPCs

B

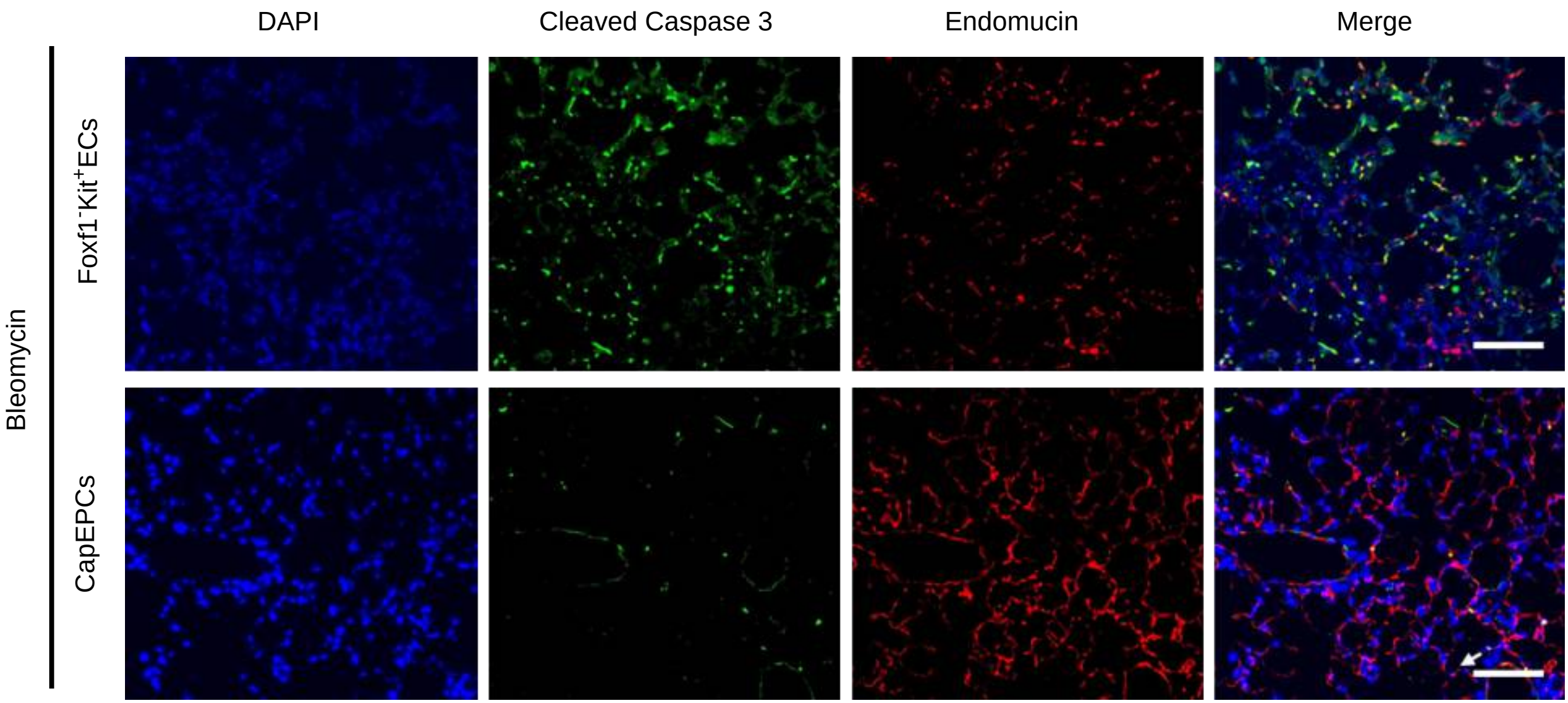
DAPI
Cleaved Caspase 3
Endomucin
Merge
Bleomycin
Foxf1⁻Kit⁺ECs
CapEPCs

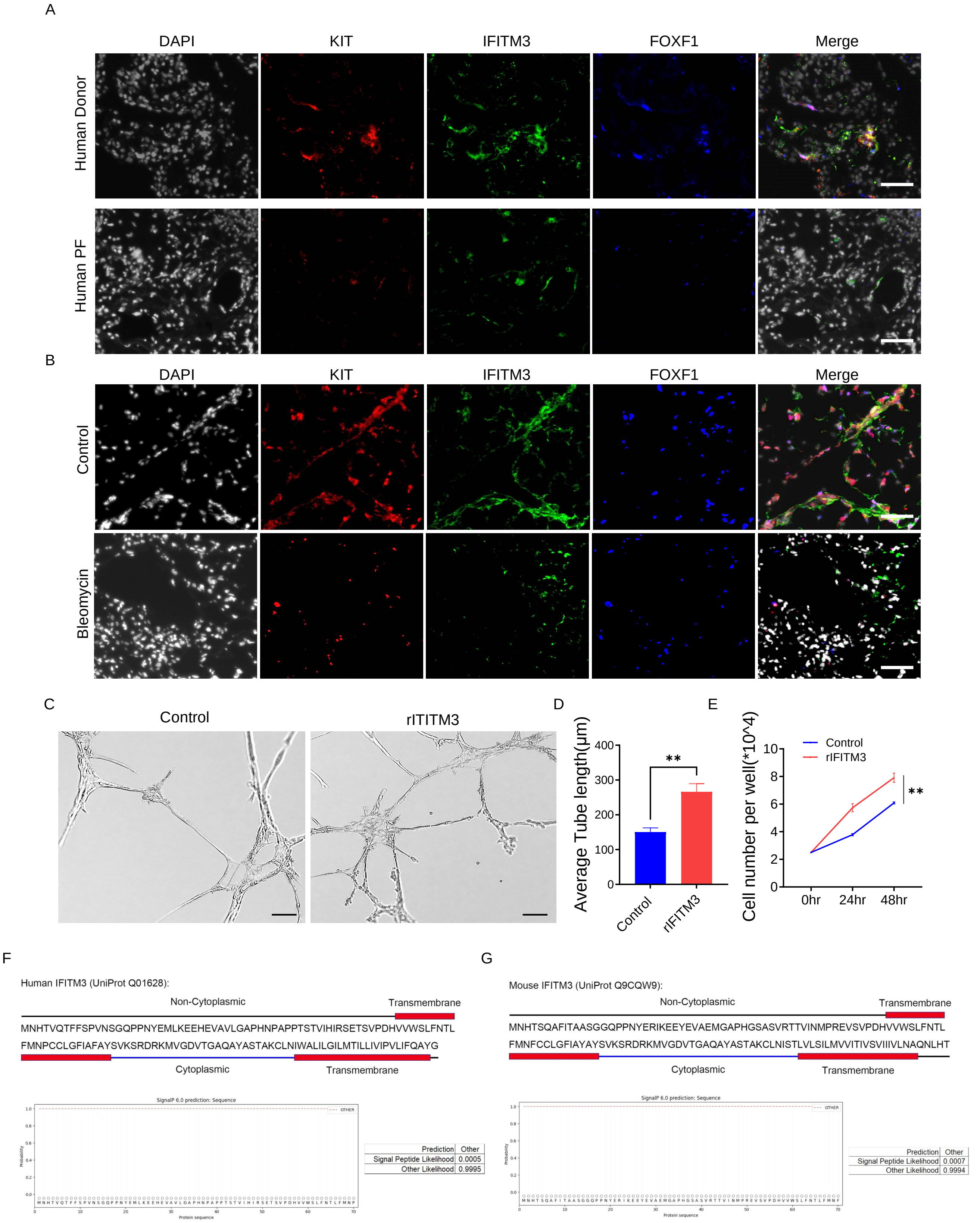
A
DAPI
KIT
IFITM3
FOXF1
Merge
Human Donor
Human PF
B
DAPI
KIT
IFITM3
FOXF1
Merge
Control
Bleomycin
C
Control
rITITM3
D
Average Tube length(μm)
**
Control
rIFITM3
E
Cell number per well(*10^4)
Control
rIFITM3
**
0hr
24hr
48hr
F
Human IFITM3 (UniProt Q01628):
Non-Cytoplasmic
Transmembrane
MNHTVQTFFSPVNSGQPPNYEMLKEEHEVAVLGAPHNPAPPTSTVIHIRSETSVPDHVVWSLFNTL
FMNPCCLGFIAFAYSVKSRDRKMVGDVTGAQAYASTAKCLNIWALILGILMTILLIVIPVLIFQAYG
Cytoplasmic
Transmembrane
SignalP 6.0 prediction: Sequence
Prediction | Other
Signal Peptide Likelihood | 0.0005
Other Likelihood | 0.9995
G
Mouse IFITM3 (UniProt Q9CQW9):
Non-Cytoplasmic
Transmembrane
MNHTSQAFITAASGGQPPNYERIKEEYEVAEMGAPHGSASVRTTVINMPREVSVPDHVVWSLFNTL
FMNFCCLGFIAYAYSVKSRDRKMVGDVTGAQAYASTAKCLNISTLVLSILMVVITIVSVIIIVLNAQNLHT
Cytoplasmic
Transmembrane
SignalP 6.0 prediction: Sequence
Prediction | Other
Signal Peptide Likelihood | 0.0007
Other Likelihood | 0.9994

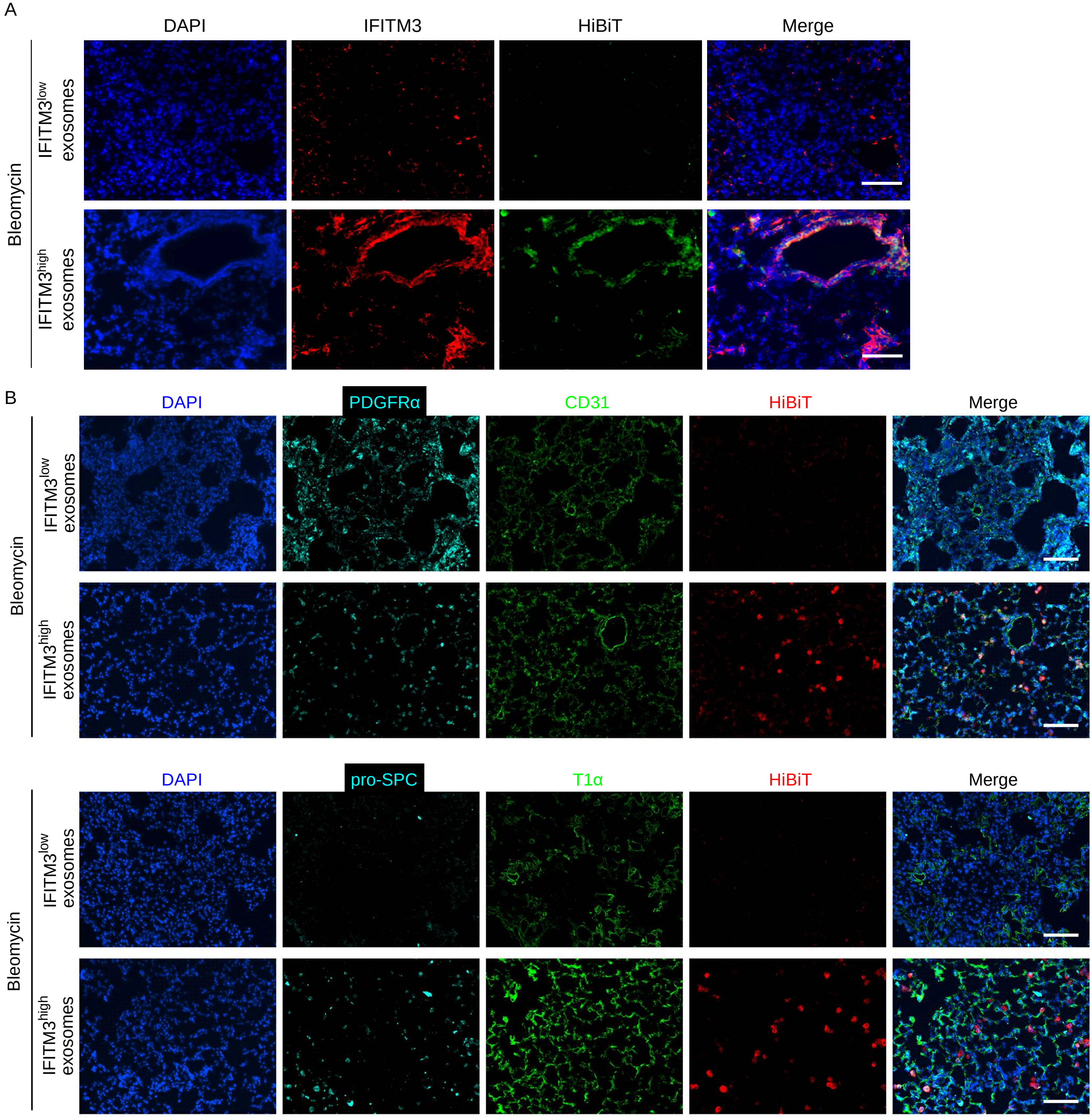
A
DAPI
IFITM3
HiBiT
Merge
Bleomycin
IFITM3low exosomes
IFITM3high exosomes
B
DAPI
PDGFRα
CD31
HiBiT
Merge
Bleomycin
IFITM3low exosomes
IFITM3high exosomes
DAPI
pro-SPC
T1α
HiBiT
Merge
Bleomycin
IFITM3low exosomes
IFITM3high exosomes

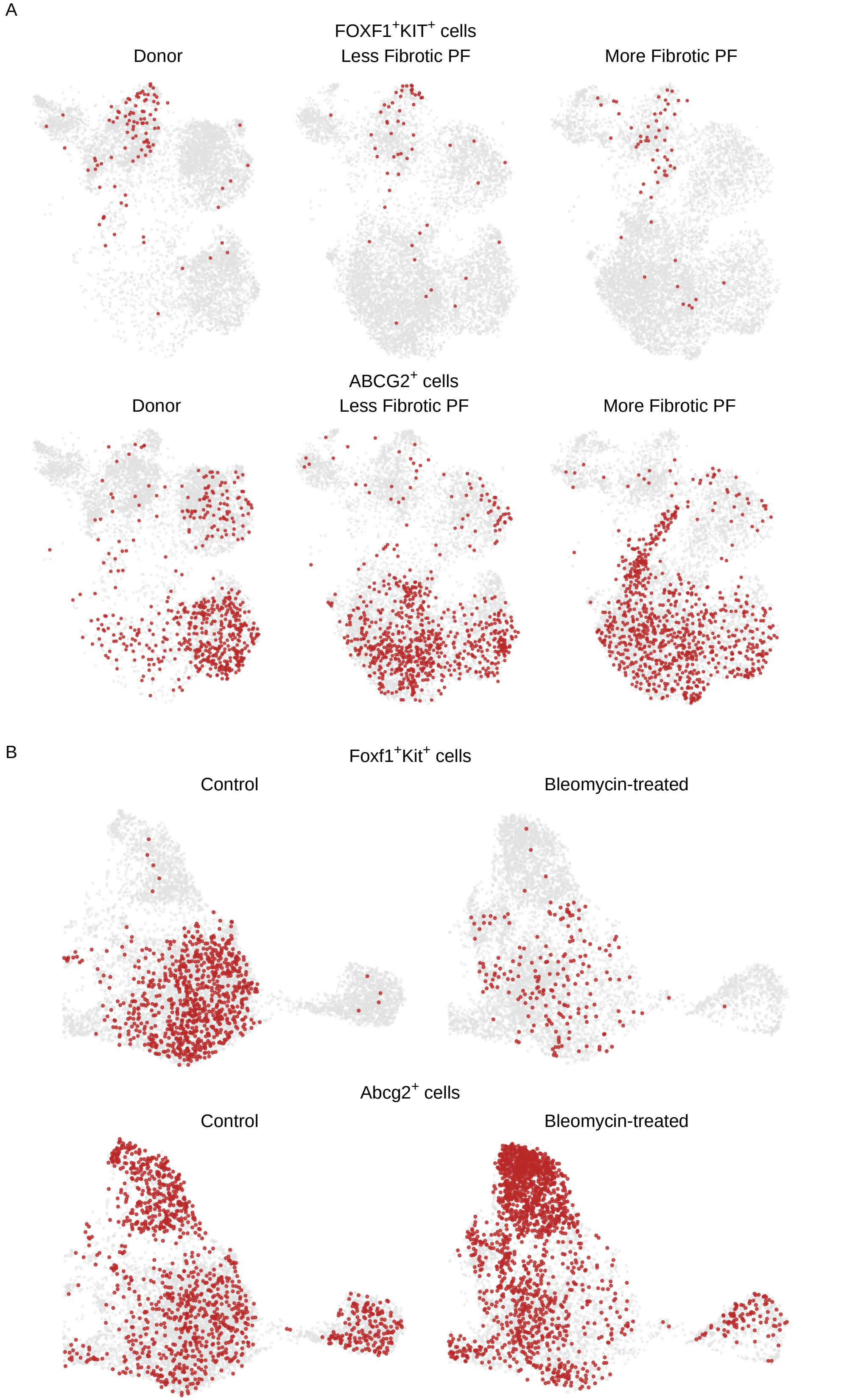
A
FOXF1^{+}KIT^{+} cells
Donor
Less Fibrotic PF
More Fibrotic PF
ABCG2^{+} cells
Donor
Less Fibrotic PF
More Fibrotic PF
B
Foxf1^{+}Kit^{+} cells
Control
Bleomycin-treated
Abcg2^{+} cells
Control
Bleomycin-treated

Table S1. Size measured by DLS in exosomes isolated from HPAECs

		Diameter (nm)			
		Mean	Standard Deviation	Minimum	Maximum
Exo Control	Peak1	94.50	11.37	86.07	107.43
IFITM3^low^exosomes	Peak1	99.73	24.31	72.28	146.37
	Peak2	17.24	6.37	7.50	27.06
IFITM3^high^exosomes	Peak1	111.47	21.88	84.11	141.61
	Peak2	22.44	4.22	17.80	29.12

Table S2. Antibodies used for Flow cytometry

Antibody	Conjugate	Company	Catalog #	Dilution
CD31	eF450	eBioscience	48-0311-82	1:100
CD45	PE-Cy7	Biolegend	103114	1:100
CD117	BV711	Biolegend	105835	1:80

Table S3. primers for cloning

Ifitm3_fwd	CCTAACTGGCCGGTACCctcttaaacactgaggtttgggcc
Ifitm3_rev	TTGATATCCTCGAGGCTAGCGAGCTCAggtgcggagcaaaggcagca
Hibit-IFITM3_Vector_fwd	GTTTAAACGGCCGCGACTC
Hibit-IFITM3_Vector_rev	GCTAATCTTCTTGAACAGCCGCC
HiBiT-IFITM3_Insert_fwd	TTCAAGAAGATTAGCCTTATGGCCATGGAGGCCCG
HiBiT-IFITM3_Insert_rev	CGCGGCCGTTTAAACCTATCCATAGGCCTGGAAGATCAGC

Table S4. List of Taqman probes

Mouse Taqman Probes for qRT-PCR			
Foxf1	Mm00487497_m1	*Pecam1*	Mm01242576_m1
Col1a1	Mm00801666_g1	*Cdh5*	Mm00486938_m1
Col3a1	Mm00802333_m1	*Kdr*	Mm01222421_m1
Fn1	Mm01256744_m1	*Ifitm3*	Mm00847057_s1
Acta2	Mm00725412_s1	*Actb*	Mm02619580_g1
Vim	Mm00449201_m1		
Human Taqman Probes for qRT-PCR			
COL3A1	Hs00943809_m1	*VIM*	Hs00958111_m1
ACTA2	Hs00426835_g1	*FN1*	Hs01549976_m1
ACTB	Hs01060665_g1		

Table S5. Antibodies used for immunofluorescence

Antibody	Company	Catalog #	Dilution
Foxf1	R&D Systems	AF4798	1:100
CD31	Novus biologicals	NB600-1475	1:100
CD31	R&D Systems	AF3628	1:100
αSMA	Sigma Aldrich	A5228	1:3000
cKit	Cell signaling Technology	3074S	1:100
HiBiT	Promega	N7200	1:200
T1α	DSHB	8.1.1	1:100
Pro-SPC	Seven Hills	WRAB-9337	1:100
PDGFRα	Thermo Fisher Scientific	MA5-41209	1:200
IFITM3	abcam	ab15592	1:200
Ki67	BD Pharmingen™	5506091:	1:100
Endomucin	abcam	AB106100	1:100
cleaved caspase-3	Cell Signaling Technology	9661	1:100
CTHRC1	Thermo Fisher Scientific	16534-1-AP	1:100
